# Versatile Enzymatic Approach for Truncation, Labeling, and Mutation of Nucleobase‐Modified Aptamers Demonstrated on Indole‐Modified *β*‐Conglutin Aptamer

**DOI:** 10.1002/cbic.202500534

**Published:** 2025-09-26

**Authors:** Marek Ondruš, Pablo Alberto Franco‐Urquijo, Teresa Mairal Lerga, Lucie Mužíkova Čechová, M. Carmen Bermudo Redondo, Ciara K. O´Sullivan, Michal Hocek

**Affiliations:** ^1^ Institute of Organic Chemistry and Biochemistry Czech Academy of Sciences Flemingovo nam. 542/2 160 00 Prague 6 Czech Republic; ^2^ Interfibio Consolidated Research Group Departament d’Enginyeria Química Universitat Rovira i Virgili 43007 Tarragona Spain; ^3^ Institució Catalana de Recerca i Estudis Avancats (ICREA) 08010 Barcelona Spain; ^4^ Department of Organic Chemistry Faculty of Science Charles University in Prague Hlavova 8 12843 Prague 2 Czech Republic

**Keywords:** aptamers, enzymatic syntheses, nucleotides, oligonucleotides, polymerases

## Abstract

A new enzymatic method for truncation of aptamers from 5’‐end has been developed and demonstrated on a newly selected indole‐modified *β*‐conglutin aptamer. This method relies on extension of a primer containing ribonucleotides, which can be specifically hydrolyzed by RNase, thereby removing the whole or 5’‐part of the primer. This approach enables flexible synthesis of modified aptamers truncated from the 5’ end, 3’ end, or both ends—a capability previously achievable only through chemical oligonucleotide synthesis. Furthermore, by employing doubly labeled primers, diverse labeled or modified nucleotides are introduced to prepare 5’‐labeled aptamers suitable for fluorescence‐ or immobilization‐based assays. The different variations of this method also enable the synthesis of mutated sequences to study the effect of modifications and their positions. Overall, this enzymatic method provides a valuable alternative for the truncation or labeling of (hyper)modified and functional oligonucleotides avoiding the need for chemical synthesis.

## Graphical abstract

Enzymatic methodology for truncation of base modified aptamers from 3’‐, 5’‐ or 3’‐ and 5’‐ends suitable also for 5’‐labeling or partially mutated versions, is presented and exemplified on a *β*‐conglutin aptamer containing indole modifications.

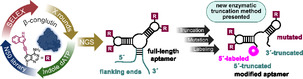

## 1 Introduction

Natural nucleic acids are biopolymers responsible for storing and propagating genetic information. In addition to their canonical roles, they are highly versatile molecules with regulatory, ligand‐binding, and catalytic capabilities.^[^ ^1^ ^]^ Single‐stranded sequences that are folded into defined secondary structures and bind with high affinity and specificity to their targets are called aptamers and are selected by systematic evolution of ligands by exponential enrichment (SELEX) from oligonucleotide libraries.^[^ ^2^, ^3^ ^–^ ^4^ ^]^ These nucleic acid ligands, often rivaling affinity and specificity of antibodies, can be selected against various clinically or diagnostically relevant targets—from small molecules^[^ ^5^, ^6^ ^–^ ^7^ ^]^ to proteins^[^ ^8^, ^9^, ^10^, ^11^, ^12^ ^–^ ^13^ ^]^ or whole cells.^[^ ^14^ ^]^ Unlike conventional antibodies produced by immune responses, aptamers can be chemically or enzymatically synthesized with high purity, eliminating batch‐to‐batch variability and allowing for scalable production.^[^ ^15^ ^,^ ^16^ ^]^ Consequently, aptamers have been utilized as high‐affinity molecular recognition elements in a variety of sensor formats for clinical and food safety diagnostic tools.^[^ ^15^ ^,^ ^17^ ^]^

Nucleobase‐modified aptamers expand the chemical diversity of aptamers beyond the four canonical bases and enrich the binding interface with functionalities like those found in antibodies. Aromatic and hydrophobic moieties that mimic amino acid side chains directly contribute to target binding by forming additional hydrophobic contacts, *π–* *π* stacking, or hydrogen bonds, thereby enhancing molecular recognition.^[^ ^10^ ^,^ ^18^ ^,^ ^19^ ^]^ Gold et al. have demonstrated that nucleotides extended with hydrophobic moieties significantly increase the binding affinity and stability of slow off‐rate modified aptamers (SOMAmers).^[^ ^20^ ^,^ ^21^ ^]^ Similarly, modified aptamers with indole, cubane or other functional groups attached via click chemistry have shown improved kinetics and specificity.^[^ ^22^ ^,^ ^23^ ^]^

One of the key considerations in developing aptamer‐based platforms is optimization of the length of the aptamer sequence. Aptamers are typically 40–100 nucleotides long, but not all nucleotides are necessary for target recognition.^[^ ^24^ ^]^ Regions of the aptamer that do not participate in binding can introduce unnecessary flexibility or steric hindrance. Truncating these nonessential nucleotides, such as residual primer‐binding sequences from the original library's design or other flanking domains, can improve binding affinity, specificity, and reduce the production costs of the aptamer.^[^ ^25^ ^]^ Furthermore, shortening the aptamer length can enhance sensitivity in aptamer‐based assays by affecting signal conduction,^[^ ^26^ ^,^ ^27^ ^]^ increasing structural stability, and facilitating easier incorporation into high‐density sensor formats.^[^ ^15^ ^]^ However, it is crucial to note that an aggressive shortening can destroy the secondary structure and hamper the aptamer's function. When critical nucleotide positions are removed, the aptamer may fail to fold into its binding secondary structure and suffer a severe loss of affinity or specificity.^[^ ^28^ ^]^

Construction and testing of truncated versions of unmodified aptamers is straightforward because any nucleotide position can be systematically deleted or substituted, and chemically synthesized by commercial suppliers. However, most nucleobase modifications are not commercially available and must be introduced using chemical or polymerase‐based approaches.^[^ ^29^ ^]^ Although both methods have limitations, the chemical synthesis of many variations of aptamer sequences is material‐demanding and some modifications may not be compatible with the phosphoramidite chemistry and need orthogonal protection, and hence enzymatic synthesis is more suitable for initial screening of multiple aptamer candidates.^[^ ^30^ ^]^

**Scheme 1:**
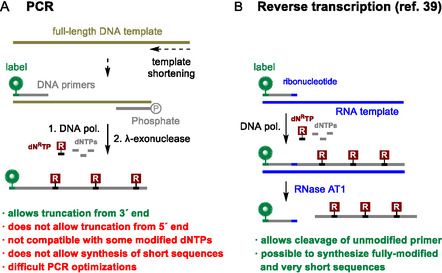
A) PCR allowing truncation from 3′ end and B) RT method using ribonucleotide‐containing primer for removal of unmodified primer.

PCR, a technique widely employed by researchers to produce modified aptamer candidates, only allows for truncation from the 3'‐end by shortening the original DNA template (**Scheme** **1**A). Possible PCR truncation from 5'‐end using a different primer would introduce a nonmodified sequence that may hamper the proper binding to the target. Therefore, so far, the chemical synthesis was the only option for 5'‐truncation of modified aptamers. PCR also does not provide exponential amplification when multiple modified dNTPs are used at the same time; hence the production of hypermodified aptamers is also not possible.^[^ ^31^ ^]^ Therefore, truncation of modified aptamers from the 5'‐end by enzymatic methods still remains an unsolved challenge. Several other enzymatic approaches have been employed to synthesize shorter modified oligonucleotides. Nicking enzyme amplification reaction (NEAR) was used for production of short base‐modified oligonucleotides,^[^ ^32^ ^]^ while Lovelock group reported a similar enzymatic cascade that enables oligonucleotide production with modifications at the 2′ position of sugar or within the phosphate moiety.^[^ ^33^ ^]^ Exonuclease mapping^[^ ^34^, ^35^ ^–^ ^36^ ^]^ and in silico structure‐guided predictions methods^[^ ^37^ ^,^ ^38^ ^]^ became popular, but all require a postsynthetic labeling step with additional validation. Recently, we published a reverse transcription (RT)‐based method using an RNA template and a ribonucleotide‐containing primer to remove the primer region of hypermodified DNA (Scheme 1B).^[^ ^39^ ^]^ In this method, after RT catalyzed by engineered DNA polymerases, RNase AT1 digests the RNA template and specifically recognizes and cleaves ribonucleotide positions within the primer, thereby enabling the complete removal of the unmodified primer. We envisaged that variations of this concept can be used as a new method for the production of modified truncated aptamers.

## 2 Results and Discussion

As previously mentioned, enzymatic methods for producing truncated versions of nucleobase‐modified aptamers are highly needed and we established the development of a methodology to address this need. We decided to develop and demonstrate it on the selection and truncation of a new indole‐modified aptamer against *β*‐conglutin. The indole‐modified **dA** ^**EIn**^ **TP** was chosen for its potential to improve aptamer binding affinity by forming additional hydrophobic interactions, *π–* *π* stacking, or hydrogen bonding. *β*‐conglutin can produce anaphylactic shock in susceptible individuals, while white lupin flour is widely utilized as a high‐protein, gluten‐free ingredient in bread, pasta, and various food products.^[^ ^40^ ^,^ ^41^ ^]^ Although the European Union now mandates clear labeling of all lupin‐containing ingredients, sensitive detection of *β*‐conglutin remains crucial due to the potential presence of hidden lupin in foods.^[^ ^42^, ^43^ ^–^ ^44^ ^]^ We recently developed two DNA aptamers binding *β*‐conglutin with nanomolar affinities.^[^ ^45^, ^46^ ^–^ ^47^ ^]^ These unmodified aptamers were successfully integrated into an isothermal amplification‐based assay^[^ ^48^ ^]^ as well as rapid lateral flow assays based on competitive formats^[^ ^49^ ^,^ ^50^ ^]^ with high sensitivity, demonstrating that aptamer‐based detection of lupin allergens is feasible. However, as with many aptamers selected from natural nucleic acid libraries, the performance of the *β*‐conglutin aptamer could be further improved, or dual‐binding aptamers could be selected to facilitate robust sandwich assays.

**Scheme 2:**
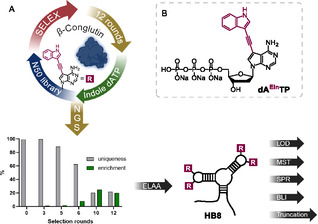
A) Graphical representation of the selection process for the identification of **HB8** aptamer. B) With chemical structure of indole‐modified dATP.

The initial single‐stranded DNA (ssDNA) library for SELEX consisted of fifty randomized positions flanked by two constant primer‐binding sites. *β*‐conglutin was immobilized on magnetic beads, and its presence was confirmed by a qualitative binding assay using a previously published *β*‐CBA‐I aptamer (Figure S1, Supporting Information). The library was incubated with the *β*‐conglutin‐functionalized beads, and bound sequences underwent PCR using 5'‐phosphorylated reverse primer and previously reported indole‐modified adenosine triphosphate (**dA** ^**EIn**^ **TP**) (**Scheme** **2**, Section S3, Supporting Information).^[^ ^31^ ^]^ The resulting double‐stranded DNA (dsDNA) was subjected to *λ*‐exonuclease digestion, yielding an indole‐modified single‐stranded library used in further selection rounds (Figure S4, Supporting Information). Aptamer selection consisted of twelve selection rounds (Section S4.1, Supporting Information). The enrichment of binding sequences was monitored through the amount of ssDNA recovered and amplified in PCR (Figures S5−6, Supporting Information). Next‐generation sequencing (NGS) analysis indicated the enrichment of binding sequences and a decreasing percentage of unique sequences as the number of rounds increased, confirming the evolution of the indole‐modified pools (Table S3 and S4, and Figures S7–9, Supporting Information). Eight aptamer candidates were selected based on their frequencies (Figure S8, Supporting Information) and were produced by PCR and *λ*‐exonuclease digestion (Figures S11−13, Supporting Information). After purification (Figure S14, Supporting Information), all candidates were evaluated with an Enzyme‐linked aptamer assay (ELAA) for binding to *β*‐conglutin (Figures S15−16, Supporting Information). Based on the obtained affinities, the best‐binding aptamer (**HB8**, K_D_ = 0.6 nM) was further tested for cross‐target specificity (Figure S18, Supporting Information), limit of detection (LOD, Figure S19, Supporting Information) and its binding affinity was additionally validated by surface plasmon resonance (SPR) (Figure S20, Supporting Information), microscale thermophoresis (MST) (Figures S21−22, Supporting Information), and biolayer interferometry (BLI) (Figures S23−25, Supporting Information).

**Scheme 3:**
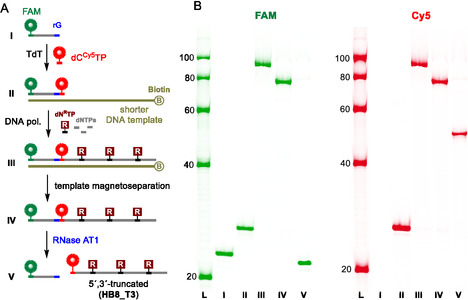
A) PEX‐based method allowing for 5' end truncation and labeling and B) the corresponding FAM and Cy5 scans of PAGE analysis: (I) Fw_rG primer; (II) TdT labeling reaction, (III) crude PEX reaction, (IV) magnetoseparation, and (V) RNase cleavage for **HB8_T3** production.

The newly selected indole‐modified aptamer was utilized to develop a novel enzymatic method for truncation, labeling, and production of mutated sequences. The approach builds on our previously published RT‐based method, which employed an RNA template and a DNA primer with a ribonucleotide (rN) at the end of the sequence (Scheme 1B).^[^ ^39^ ^]^ In this method, RNase AT1 digested the RNA template into single nucleotides removable by spin columns, recognized the rN position within the primer, and allowed for removal of unmodified primer. RNase AT1 is a mixture of two enzymes. RNase A specifically recognizes and hydrolyzes rC and rU positions, while RNase T1 hydrolyzes RNA at rG residues, providing high variability in the positioning of the rN within the primer. Once the primer bearing the label (e.g., fluorophore) is cleaved, the extended modified sequence becomes invisible on the gel and cannot be directly utilized in any fluorescence‐ or immobilization‐based assays. To overcome this disadvantage and produce a truncated aptamer with the 5'‐end label, we separately prepared a doubly‐labeled primer using terminal transferase (TdT) and fluorescently‐labeled **dC** ^**Cy5**^ **TP** (**Scheme** **3**A, Figures S26−27, Supporting Information). This resulted in the extension of the primer by one nucleotide and the fluorescent label behind the cleavable ribonucleotide. Furthermore, due to the high cost of RNA sequences and their instability, the RNA template and its digestion step were replaced by a biotinylated DNA template and conventional magnetoseparation using streptavidin‐coated magnetic beads^[^ ^31^ ^]^. Therefore, primer extension (PEX) reaction was performed instead of RT. This configuration enabled the production of 5′‐Cy5‐labeled **HB8_T3** aptamer truncated from both the 5´ and 3´ ends (Scheme 3B, Figure S30, Supporting Information).

**Scheme 4:**
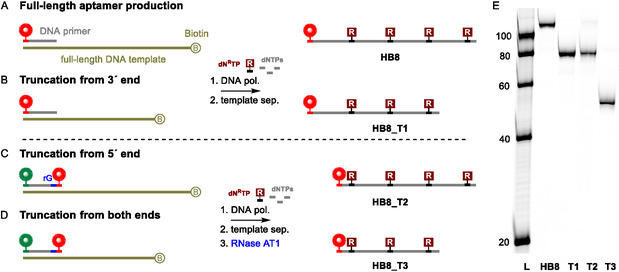
A–D) The newly presented PEX‐based method enables flexible truncations and E) Cy5 scan of PAGE analysis.

To demonstrate the method's versatility, we completed a whole series of **HB8** aptamers truncated from the 3’‐end (**HB8_T1**), 5’‐end (**HB8_T2**), or both ends (**HB8_T3**) (**Scheme** **4**A, **Table** **1**). All three aptamer truncations, along with the full‐length aptamer, were analyzed on a polyacrylamide gel (Scheme 4B, Figure S31, Supporting Information) and characterized by LC‐MS (Figures S34−49, Supporting Information). As shown in our previously published work,^[^ ^39^ ^]^ this method alseo allows the synthesis of fully modified and very short (<20 nt) sequences. One could consider labeling the truncated aptamers at the 3'‐end using the TdT. However, all truncated versions would need to undergo the labeling reaction to have a label at the same end. In our approach, one primer can be used for various truncated versions.

**Table 1:** Overview of the synthesized truncated (T) and mutated (M) versions of **HB8**. The red‐colored A stands for the indole‐modified position.

Aptamer	Sequence
**HB8**	AGCTCCAGAAGATAAATTACAGGCCAAGATCTCGCGAGTTCCCAACAGGGCAGAGAGATGCCCGACACCAGTGCAACTAGGATACTATGACCCC
**HB8_T1**	AGCTCCAGAAGATAAATTACAGGCCAAGATCTCGCGAGTTCCCAACAGGGCAGAGAGATGCCCGACACCAGTG
**HB8_T2**	CCAAGATCTCGCGAGTTCCCAACAGGGCAGAGAGATGCCCGACACCAGTGCAACTAGGATACTATGACCCC
**HB8_T3**	CCAAGATCTCGCGAGTTCCCAACAGGGCAGAGAGATGCCCGACACCAGTG
**HB8_T4**	GATAAATTACAGGCCAAGATCTCGCGAGTTCCCAACAGGGCAGAGAGATGCCCGACACCAGTGCAACTAGGATACTATGACCCC
**HB8_M1**	AGCTCCAGAAGATAAATTACAGGCCAAGATCTCGCGAGTTCCCAACAGGGCAGAGAGATGCCCGACACCAGTGCAACTAGGATACTATGACCCC
**HB8_M2**	AGCTCCAGAAGATAAATTACAGGCCAAGATCTCGCGAGTTCCCAACAGGGCAGAGAGATGCCCGACACCAGTGCAACTAGGATACTATGACCCC

**Scheme 5:**
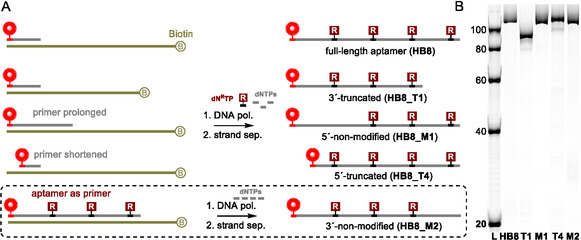
A) Schematic representation for production of truncated and mutated **HB8** aptamers and B) Cy5 scan of PAGE analysis.

Removing small parts of the sequence or replacing modified nucleotides with their nonmodified counterparts can provide insights into key target recognition positions. To demonstrate the flexibility of the method, we used longer and shorter DNA primers or employed aptamer **HB8_T1** as a primer in the PEX and synthesized mutated sequences (**Scheme** **5**). Shortening the primer while maintaining a reasonable melting temperature compatible with the extension reaction can effectively eliminate the very first unmodified primer nucleotides. We used 10 nt shorter primer and produced **HB8_T4** as a representative of this option. On the other hand, prolonging the primer over the initial modified positions enabled us to produce mutant **HB8_M1**. Such mutant sequences allow probing of the importance of central modified positions. Aptamer **HB8_T1** was also used as a primer in PEX with natural dNTPs. Such synthesis yielded a mutant **HB8_M2** that was unmodified at its 3’‐end (Figure S32, Supporting Information).

With this, we demonstrated that virtually every truncation from the 5'‐end, 3'‐end, or both ends of the **HB8** aptamer was feasible by using an appropriate template and labeled primer containing the ribonucleotide. Moreover, we showed that primer length compatible with the extension reaction conditions allows the production of mutant sequences that can be used to probe the effect of modifications.

**Figure 1:**
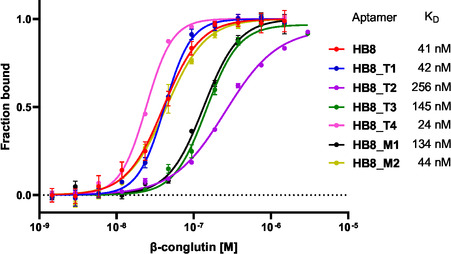
MST measurements of full‐length **HB8** aptamer and its truncated and mutated versions with their calculated K_D_ values. Error bars represent the mean ± s.d. from three technical replicates. Dissociation constants (K_D_) were calculated using the GraphPad Prism software using a specific binding with Hill slope model nonlinear regression model.

Subsequently, we measured the binding affinities of all four truncated and both mutated aptamers using MST to investigate the importance of each primer region and the indole moiety for target recognition (**Figure** **1**, Figure S21, Supporting Information). The 3´‐truncated **HB8_T1** showed a binding affinity of 42 nM, similar to the original full‐length **HB8**'s affinity (41 nM), indicating that the removed 21‐nucleotide region does not participate in target binding. When indole‐modified nucleotides within this region were replaced with their natural counterparts (**HB8_M2**), the binding affinity remained very similar (44 nM). These results indicate that removing part of the 3'‐end region (complementary to the primer in the original library) or replacing the indole‐modified nucleotides with their natural counterparts in this region does not have a negative effect on the affinity. It was further confirmed by measurement of **HB8** and **HB8_T1** aptamers using BLI and ELAA that showed similar K_D_ values (Figures S17, S24, and S25, Supporting Information). The K_D_ values calculated from MST were higher compared to values from BLI and ELAA. Similar differences between MST and BLI methods have been previously described for some aptamer‐target interactions where interfering effects like avidity or immobilization of one binding partner altered the binding behavior.^[^ ^51^ ^,^ ^52^ ^]^ On the other hand, replacing three indole‐modified nucleotides with their natural counterparts within the central region of the full‐length aptamer (**HB8_M1**) negatively affected the binding affinity (K_D_ = 134 nM). Truncation of the whole primer region (23 nts) from the 5'‐end (**HB8_T2**) or both ends (**HB8_T3**) resulted in lower affinity aptamers (K_D_ = 256 nM and 145 nM, respectively), indicating an important contribution of at least part of the 5'‐end to the formation of secondary structure elements necessary for binding. Finally, the **HB8_T4** aptamer truncated by 10 nts from the 5’‐end showed considerably enhanced binding affinity toward *β*‐conglutin (K_D_ = 24 nM). It was the only truncated version that enhanced the affinity to the target, assuming that the first ten nucleotides of the primer may cause some steric hindrance or unproductive secondary structure, and hence their removal improved the binding.

**Scheme 6:**
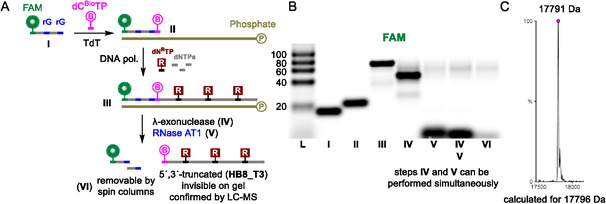
A) Schematic representation of 5'‐biotinylated **HB8_T3** production, B) corresponding FAM scan of agarose gel, and C) its MS characterization.

Many methods, including BLI and SPR, require immobilization of one of the binding partners to the surface of the platform. Not all target (bio)molecules can be easily produced with tags and hence the aptamer is typically the molecule attached to the surface. Thiol or biotin moieties, known for excellent specificity and stability, are widely used for the immobilization. Our method presented above already utilizes the biotinylated template and thus, a biotinylated primer cannot be used at the same time. To address this, the 5´‐biotinylated template can be replaced with a 5´‐phosphorylated template which is readily digested by *λ*‐exonuclease following completion of the PEX reaction (**Scheme** **6**). We used a DNA primer containing two ribonucleotides, one in the middle and the 3’‐end of the sequence, for TdT labeling with biotinylated nucleotide **dC** ^**Bio**^ **TP**. Since RNase AT1 and *λ*‐exonuclease have the same optimum temperature, it allowed the digestion of the 5´‐phosphorylated template and ribonucleotide cleavage to occur during the same incubation period or separately if needed. Once the primer was cut into short fragments, spin column purification effectively removed these fractions, affording the 5´‐biotinylated **HB8_T3** aptamer in pure and desalted form ready for BLI measurements. For longer DNA primers, it is recommended to use two to three ribonucleotide positions to cleave them into shorter fragments that are more effectively removable by spin columns than their longer counterparts.

**Figure 2:**
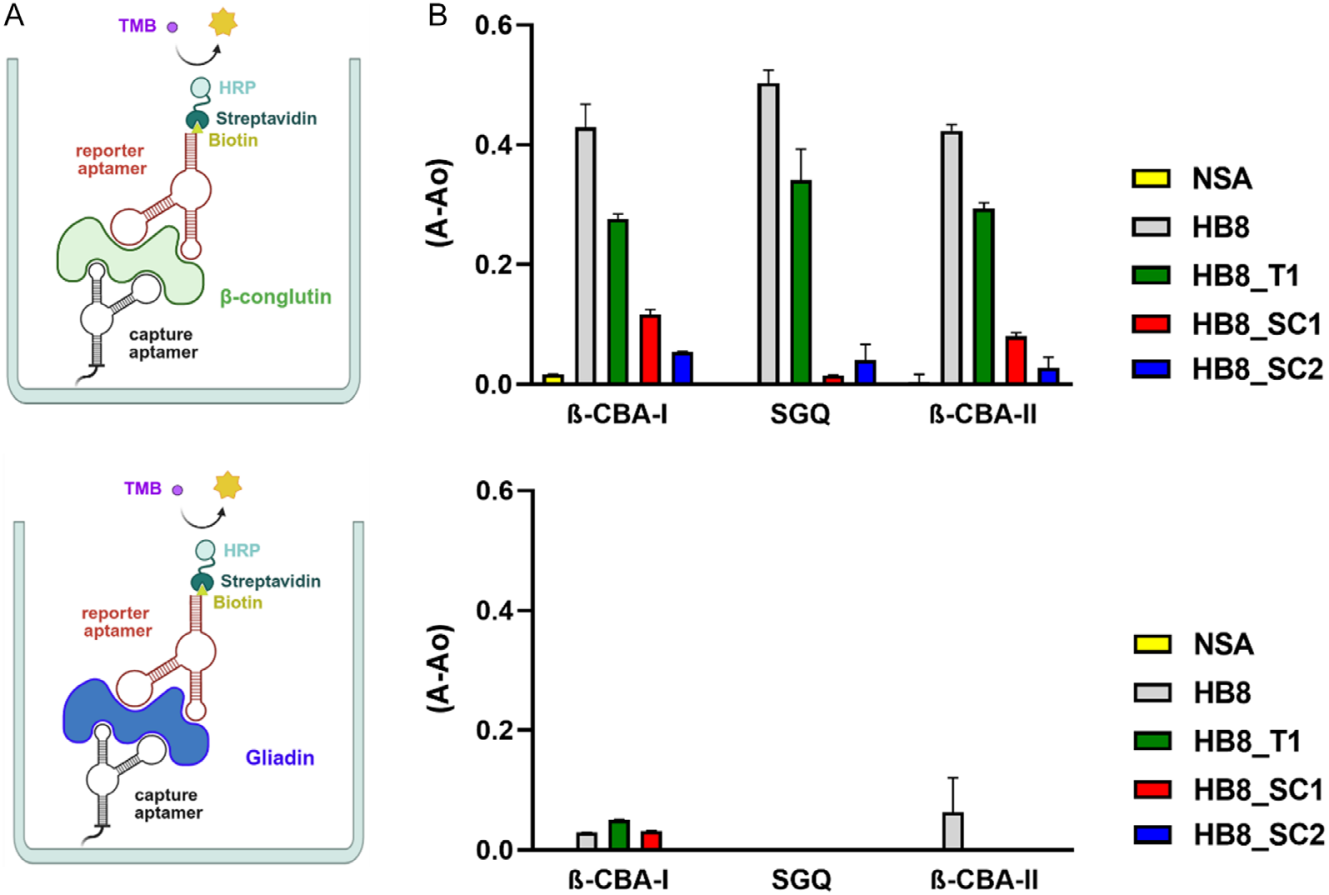
A) Plate‐based sandwich assay for *β*‐conglutin and gliadin with unmodified aptamers immobilized as capture probes and indol‐modified aptamers bound to the target as reporter probes. B) ELAA sandwich assay for *β*‐conglutin and gliadin using **HB8**, **HB8_T1**, **HB8_SC1**, **HB8_SC2** and **NSA** as reporter probes and *β*‐CBA‐I, **SGQ**, and ***β*‐CBA‐II** as capture probes for immobilization to the plate. Error bars represent the mean ± s.d. from three technical replicates.

Both full‐length **HB8** and truncated **HB8_T1** aptamers were used in a competitive ELAA assay to determine the LOD and were compared with our previously published ***β*‐BCA‐II** aptamer (Supporting Information, Figure S19). Both **HB8** and **HB8_T1** aptamers showed similar LODs, 166 pM and 118 pM, respectively, compared to 273 pM obtained from the unmodified ***β*‐BCA‐II** aptamer. More importantly, we assumed that indole‐modified aptamers would preferably bind to the hydrophobic region of *β*‐conglutin. Along with ***β*‐CBA‐II** or other unmodified aptamers like ***β*‐CBA‐I** and **SGQ** binding to hydrophilic aptatopes, this approach helped us develop a robust plate‐based sandwich assay (**Figure** **2**A). We immobilized 5´‐thiolated ***β*‐CBA‐I**, **SGQ**, and ***β*‐CBA‐II** aptamers on maleimide‐functionalized plates. Following the complex formation with *β*‐conglutin, indole‐modified **HB8**, truncated (**HB8_T1**), scrambled sequences (**HB8_SC1** and **HB8_SC2**), and a nonspecific aptamer (**NSA**) were added. Both modified aptamers successfully formed a sandwich for ***β*‐conglutin** but not for gliadin (protein control), confirming our hypothesis about simultaneous binding and different binding aptatopes for modified and unmodified aptamers (Figure 2B). The scrambled sequences **HB8_SC1** and **HB8_SC2** showed some minimal binding with ***β*‐CBA‐I** and ***β*‐CBA‐II** capture aptamers, and the assay with **SGQ** capture aptamer was very specific. Using unmodified aptamers (particularly **SGQ**) as capture probes and modified aptamers as reporter probes enabled us to develop a sensitive method for the detection of *β*‐conglutin.

## 3 Conclusion

We have developed a general and versatile enzymatic methodology for the synthesis of truncated and labeled aptamers. We demonstrated the methodology on an indole‐modified aptamer against *β*‐conglutin. We synthesized an indole‐modified DNA library and used it for twelve consecutive selection rounds to isolate a modified *β*‐conglutin aptamer with nanomolar affinity. It simultaneously binds the target together with previously published unmodified aptamers. Based on this, we developed a plate‐based sandwich assay for sensitive detection of *β*‐conglutin. Additionally, we investigated the importance of its 5´ and 3´ regions using a new enzymatic truncation method and MST. The truncation approach is based on PEX of a DNA primer containing a ribonucleotide at the 3’‐end, template magnetoseparation, and RNase cleavage to remove the 5’‐end primer region to afford 5´‐truncated nucleobase‐modified aptamers. Using a primer with two ribonucleotides (one in the middle and one at the 3’‐end), the cleavage by RNase gives short oligonucleotide fragments easily removable by spin columns, thus facilitating the purification of the truncated aptamers. The methodology can be also used for generation of 5’‐labeled truncated aptamers containing a fluorophore or biotin when using single nucleotide extension with labeled dNTP and 5´‐phosphorylated templates that are readily digested by *λ*‐exonuclease. We demonstrated the methodology on base‐modified aptamers, but in principle it could be used for other types of modifications including sugar‐modified XNA, for example (3′, 2′)‐*α*‐l‐threose nucleic acid (TNA),^[^ ^53^ ^]^ 2′‐O, 4′‐C‐methylene‐*β*‐D‐ribonucleic acid (LNA),^[^ ^54^ ^]^ 2′‐fluoro‐^[^ ^55^ ^]^ or 2′‐O‐alkyl^[^ ^56^ ^]^ modifications, if appropriate engineered polymerases will be used for the PEX and proper optimization of all the step is performed. It should be noted that alternative ways for the removal of 5’‐primer based on incorporation of either 2’‐deoxyinosine or 2’‐deoxyuridine and cleavage by EndoV endonuclease^[^ ^33^ ^]^ or uracil‐DNA glycosylase^[^ ^57^ ^]^ could also be considered in the future but would require a thorough development and optimization.

This method extends a repertoire of modified truncated and mutated sequences, providing the necessary tools to probe the modified aptamer by the length and position of modifications, thus eliminating the need for chemical synthesis in the early stages of aptamer optimization which now can be conducted exploiting enzymatic methodology using the same modified dNTPs that are used for the SELEX. However, for scaleup of synthesis of already optimized aptamers, solid‐phase chemical synthesis using the corresponding modified phosphoramidites^[^ ^58^ ^]^ still has its advantages. Alternatively, a recently reported^[^ ^59^ ^]^ milligram‐scale PEX methodology could be used for scaling up to hundreds nanomolar quantities of aptamers.

## 4 Experimental Section

4.1

4.1.1

##### PEX

Reaction mixture (20 µL) contained aptamer candidate DNA template (100 µM, 2.5 µL), 5´‐FAM‐ or Cy5‐labeled primer (100 µM, 2 µL), dNTP mix containing **dA** ^**EIn**^ **TP** (4 mM, 2.5 µL), KOD XL DNA polymerase (2.5 U) and reaction buffer (10X, 2 µL) as supplied by the manufacturer. The reaction mixture was incubated for two hours at 60 °C and then stopped by cooling to 4 °C.

##### Magnetoseparation

C1 magnetic Dynabeads (150 µL) were used for one PEX (20 µL) reaction. Beads were washed with 1X binding buffer (5 mM Tris‐HCl, 0.5 mM EDTA, 1 M NaCl, pH 7.5) (3x 400 µL) and incubated with diluted PEX reaction (20 µL of PEX reaction + 380 µL of water + 400 µL of 2X binding buffer). The mixture was incubated for 1 h at room temperature on a Hulamixer. Beads were washed with binding buffer (3x 400 µL), and 50 µL of 20 mM NaOH was added, vortexed, and incubated for 10 min at room temperature using a Hulamixer. Beads were attached to a magnet, and supernatant (≈44 µL) containing ssDNA was taken. KOD XL buffer (10X, 5 µL) was added, followed by HCl (0.5 M, 1 µL) neutralization. Samples containing rNs within the primer region (50 μL after magnetoseparation) were incubated with RNase AT1 (5 U) for 8 h at room temperature.

##### Simultaneous RNase Digestion

Reaction mixture (50 µL) contained crude PEX (20 µL), *λ*‐exonuclease (10 U), RNase AT1 (10 U), and KOD XL buffer (10X, 4 µL). The reaction mixture was incubated at 37 °C for 12 h to achieve complete digestion.

## Supporting Information

Supporting Information is available containing detailed experimental part, additional gels, tables and figures, as well as copies of MS spectra.

## Conflict of Interest

The authors declare no conflict of interest.

## Author Contributions

**Marek Ondruš** and **Pablo Alberto Franco‐Urquijo** designed the truncation method, performed the synthesis and testing of truncated and modified aptamers and wrote the manuscript with contribution of all other authors. **Teresa Mairal Lerga** performed SELEX, SPR, and ELAA‐based experiments. **Lucie Mužíková Čechová** measured BLI. **Marek Ondruš** measured LC‐MS. **Pablo Alberto Franco‐Urquijo** analyzed NGS data. **M. Carmen Bermudo Redondo** performed sandwich assay. **Ciara K. O´Sullivan** and **Michal Hocek** conceptualized and supervised the study.

## Supporting information

Supplementary Material

## Data Availability

The data that support the findings of this study are available in the supplementary material of this article.
