# Supplementary material for: Versatile Enzymatic Approach for Truncation, Labeling, and Mutation of Nucleobase‐Modified Aptamers Demonstrated on Indole‐Modified β‐Conglutin Aptamer
Source: Chembiochem. 2025 Sep 26;26(20):e202500534. doi: 10.1002/cbic.202500534 (PMC12582157; doi:10.1002/cbic.202500534)
Supplement: Supplementary file 1 — Supplementary Material [file CBIC-26-e202500534-s001.pdf]

Supporting Information

Versatile enzymatic approach for truncation, labeling, and mutation of nucleobase-modified aptamers displayed on indole-modified β-conglutin aptamer

Marek Ondruš, Pablo Alberto Franco-Urquijo, Teresa Mairal Lerga, Lucie Mužíková Čechová, M.Carmen Bermudo Redondo, Ciara K. O´Sullivan* and Michal Hocek*

Table of contents

1) **Experimental section** 3

1.1 Oligonucleotides used in this study 4

1.2 Oligonucleotides synthesized in this study 5

2) **Immobilization of β-conglutin** 6

3) **Library preparation** 6

3.1 Incorporation of indole-modified $dA^{EIn}TP$ by PCR 6

3.2 Generation of single-stranded DNA 7

4) **Aptamer selection** 8

4.1 SELEX protocol 8

4.2 Next Generation Sequencing (NGS) and data analysis 10

5) **Binding affinity and specificity assays** 13

5.1 Aptamer candidate production 13

5.2 Enzyme-linked aptamer assay (ELAA) 15

5.2.1 Cross-target selectivity assay 17

5.2.2 Competitive assay – LOD determination 18

5.2.3 Sandwich assay – epitope duality determination 19

5.3 Surface plasmon resonance (SPR) 19

5.4 Microscale thermophoresis (MST) 20

5.5 Biolayer interferometry (BLI) 22

6) **Aptamer truncation methods** 23

6.1 Synthesis of doubly-labeled primers 23

6.2 Primer extension + Magnetoseparation + RNase cleavage 25

6.3 Simultaneous λ-exonuclease and RNase cleavage 27

7) **MS characterization** 29

8) **References** 45

1) Experimental section

Phosphate Buffered Saline (PBS) tables, Dynabeads M-270 Carboxylic Acid, Dynabeads MyOne Streptavidin C1, DreamTaq DNA polymerase, Lambda Exonuclease, KOD XL DNA polymerase, GeneRuler Low Range DNA Ladder (SM1191), NUNC MaxiSorp plates, 1-Ethyl-3-(3-dimethylaminopropyl) carbodiimide (EDC), N-hydroxysuccinimide (NHS), agarose for 3% electrophoresis gel, natural dNTPs and RNase AT1 were purchased from ThermoFisher Scientific. Terminal transferase (TdT) was purchased from New England Biolabs. Oligonucleotides were purchased from Biomers or Generi Biotech. **dA$^{\mathbf{EIn}}$TP** was synthesized as previously reported.[1] Magnesium chloride, skimmed milk, sulfuric acid, Pluronic F-127, 3,3′,5,5′-tetramethylbenzidine (TMB), ethanolamine, and carbonate–bicarbonate buffer were purchased from Merck. Streptavidin Poly-HRP80 was purchased from SDT-reagents. CM5 sensor chips were purchased from Cytiva. The absorbance spectra (450nm) were collected using a SpectraMax microplate reader (bioNova Sciences). NGS sequencing was performed by Ion Torrent (Centre of Omic Sciences, Eurecat Technology Center, Reus, Spain). LC-MS measurements were performed on an Agilent 1920 Infinity II BIO system with an MSD XT mass spectrometer equipped with an ESI ion source, using a Phenomenex Biozen 1.7 µm Oligo 50x2.1 mm column using buffers A (300 mM HFIP + 15 mM TEA in H_2O) and B (300 mM HFIP + 15 mM TEA in MeOH). Aquired spectra were deconvoluted using UniDec program.[2] Oligonucleotide concentrations were determined using NanoDrop1000 (ThermoFisher Scientific). dC^{Cy5}TP and dC^{Bio}TP were purchased from Jena Bioscience. Mili-Q water was used for all experiments. PAGE stop solution contained: 95% [v/v] formamide, 0.5 mM EDTA, 0.025% [w/v] bromophenol blue, 0.025% [w/v] SDS, and Milli-Q water. Reactions were analyzed using 12.5% denaturing polyacrylamide gel electrophoresis (dPAGE) (acrylamide/ bisacrylamide 19:1, 25% urea) in 1X TBE buffer (40 mA, 1 h) with preheating (except for reverse transcription reactions – no preheating, 25mA, 1 h). dPAGEs were analyzed by FAM or Cy5 fluorescence imaging using Typhoon FLA 9500 (GE Healthcare). Modified aptamer candidates were purified by C18 HPLC column (Waters XBridge BEH C18 2.5 µm, 4.6x150 mm, heated by SICO-300 column oven (SISw) or using MicroSpin G-50 (Cytiva), Oligo Clean &

Concentrator (Zymo Research) or ssDNA/RNA Clean & Concentrator (Zymo Research) spin columns. HPLC mobile phase pH was adjusted using an InoLab pH 720 meter. Other chemicals were of analytical grade. Binding buffer = PBS: 10 mM phosphate, 138 mM NaCl, 2.7 mM KCl, 1.5 mM $MgCl_2$, pH 7.4. PBST = PBS containing 0.05 % v/v Tween-20.

1.1 Oligonucleotides used in this study

Table S1. Oligonucleotides (ON) used in this study.

ON title	nt	Sequence (5´-3´)
**Initial_Lib**	94	AGCTCCAGAAGATAAATTACAGG-N50-CAACTAGGATACTATGACCCC
**Rev**[b]	21	GGGGTCATAGTATCCTAGTTG
**Fw**[a,c,d]	23	AGCTCCAGAAGATAAATTACAGG
**Fw_SC2**[a,d]	23	GGTAAATGGAATATAGCCCAACA
**Fw_rG**[c]	23	AGCTCCAGAAGATAAATTACAGrG
**Fw_2rG**[c]	23	AGCTCCAGAArGATAAATTACAGrG
**β-CBA-I**[a]	93	AGCTGACACAGCAGGTTGGTGGGGGTGGCTTCCAGTTGGGTTGACAATAC GTAGGGACACGAAGTCCAACCACGAGTCGAGCAATCTCGAAAT
**SGQ**[a]	11	GGTGGGGGTGG
**β-CBA-II**[a]	94	AGCTCCAGAAGATAAATTACAGGGGCCGGGGTGGCTCAGGCAAGGGGTT GACCTGTCGTAGGGATTGTTTTAACAACTAGGATACTATGACCCC
**NSA**	94	AGCTCCAGAAGATAAATTACAGGTCAACATAACAGATAGATAATTATTAATA TGAGTAAGGTACTAATACATACAACTAGGATACTATGACCCC
**HB1_temp**	94	AGCTCCAGAAGATAAATTACAGGCACAATGTCGCCGAGAGCGATCAAGTG TATGCACAGGTGGCACAACAGGGCAACTAGGATACTATGACCCC
**HB2_temp**	94	AGCTCCAGAAGATAAATTACAGGATGTGGACAAAGGGAAGAAGACACGAA CTGACCCACAGTAACGGTGTTCGCAACTAGGATACTATGACCCC
**HB3_temp**	93	AGCTCCAGAAGATAAATTACAGGGGAAGTATGCGGATAGCGAAACCGATG GCCGATCTTACACCCCGAGCACCAACTAGGATACTATGACCCC
**HB4_temp**	94	AGCTCCAGAAGATAAATTACAGGGGAAACGAGCCGTGAGAGATGTAAACC GTAACTGCCAAGATCGCCAGGCCCAACTAGGATACTATGACCCC
**HB5_temp**	94	AGCTCCAGAAGATAAATTACAGGAACGAGTGAGCGTGCTTCGCCAGACAA CCGGCGAAGACCGGAAACATTTCCAACTAGGATACTATGACCCC
**HB6_temp**	94	AGCTCCAGAAGATAAATTACAGGTACAAGTCATATGAGGAGAATGACAACC TCATGAACTGCCAAGGTAGCGCCAACTAGGATACTATGACCCC
**HB7_temp**	94	AGCTCCAGAAGATAAATTACAGGCAAGATCACCGTAGACGCGTCGAGAAG TGTGAGATCCTATGAAATGACCGCAACTAGGATACTATGACCCC
**HB8_temp**	94	AGCTCCAGAAGATAAATTACAGGCCAAGATCTCGCGAGTTCCCAACAGGG CAGAGAGATGCCCGACACCAGTGCAACTAGGATACTATGACCCC
**HB8_PEX_temp**[a,b]	94	GGGGTCATAGTATCCTAGTTGCACTGGTGTCGGGCATCTCTCTGCCCTGT TGGGAACTCGCGAGATCTTGGCCTGTAATTTATCTTCTGGAGCT
**HB8_T1_temp**[a,b]	73	CACTGGTGTCGGGCATCTCTCTGCCCTGTTGGGAACTCGCGAGATCTTGG CCTGTAATTTATCTTCTGGAGCT
**HB8_SC_temp**[a]	94	TGCTGCCGGGGTCGTTGCGTTGTGGTGTTCTCGGCGCAATGAATCGAACT TAGTTGATGCTCCGCATACGCCCTGTAATTTATCTTCTGGAGCT
**HB8_SC2_temp**[a]	73	TGCTGGAGTACTTGCCCTCGCGCTTCGGGTACGAGTTGCCGGCTTCAATG TGTTGGGCTATATTCCATTTACC

[a] 5´-biotinylated; [b] 5´-phosphorylated; [c] 5´-FAM; [d] 5´-Cy5; rG = RNA nucleotide.

1.2 Oligonucleotides synthesized in this study

Table S2. Oligonucleotides synthesized in this study.

ON title	nt	Sequence (5´-3´)
**Indole_Lib**	94	AGCTCCAGAAGATAAATTACAGG-N50-CAACTAGGATACTATGACCCC
**HB1**[a]	94	CACAATGTCGCCGAGAGCGATCAAGTGTATGCACAGGTGGCACAACAGGG
**HB2**[a]	94	ATGTGGACAAAGGGAAGAAGACACGAACTGACCCACAGTAACGGTGTTCG
**HB3**[a]	93	GGAAGTATGCGGATAGCGAAACCGATGGCCGATCTTACACCCCGAGCACC
**HB4**[a]	94	GGAAACGAGCCGTGAGAGATGTAAACCGTAACTGCCAAGATCGCCAGGCC
**HB5**[a]	94	AACGAGTGAGCGTGCTTCGCCAGACAACCGGCGAAGACCGGAAACATTTC
**HB6**[a]	94	TACAAGTCATATGAGGAGAATGACAACCTCATGAACTGCCAAGGTAGCGC
**HB7**[a]	94	CAAGATCACCGTAGACGCGTCGAGAAGTGTGAGATCCTATGAAATGACCG
**HB8**[a,d]	94	CCAAGATCTCGCGAGTTCCCAACAGGGCAGAGAGATGCCCGACACCAGTG
**HB8_T1**[a,d]	73	AGCTCCAGAAGATAAATTACAGGCCAAGATCTCGCGAGTTCCCAACAGGGCA GAGAGATGCCCGACACCAGTG
**HB8_T2**[d]	71	CCAAGATCTCGCGAGTTCCCAACAGGGCAGAGAGATGCCCGACACCAGTGC AACTAGGATACTATGACCCC
**HB8_T3**[a,d]	50	CCAAGATCTCGCGAGTTCCCAACAGGGCAGAGAGATGCCCGACACCAGTG
**HB8_T4**[d]	84	GATAAATTACAGGCCAAGATCTCGCGAGTTCCCAACAGGGCAGAGAGATGCC CGACACCAGTGCAACTAGGATACTATGACCCC
**HB8_M1**[d]	94	AGCTCCAGAAGATAAATTACAGGCCAAGATCTCGCGAGTTCCCAACAGGGCA GAGAGATGCCCGACACCAGTGCAACTAGGATACTATGACCCC
**HB8_M2**[d]	94	AGCTCCAGAAGATAAATTACAGGCCAAGATCTCGCGAGTTCCCAACAGGGCA GAGAGATGCCCGACACCAGTGCAACTAGGATACTATGACCCC
**HB8_SC1**[a,d]	94	AGCTCCAGAAGATAAATTACAGGGCGTATGCGGAGCATCAACTAAGTTCGATT CATTGCGCCGAGAACACCACAACGCAACGACCCCGGCAGCA
**HB8_SC2**[a,d]	73	GGTAAATGGAATATAGCCCAACACATTGAAGCCGGCAACTCGTACCCGAAGC GCGAGGGCAAGTACTCCAGCA
**Fw_dC**[Cy5(c)]	24	AGCTCCAGAAGATAAATTACAGrGC[Cy5]
**Fw_dC**[Bio(c)]	24	AGCTCCAGAArGATAAATTACAGrGC[Bio]

[a] 5´-biotinylated; [b] 5´-phosphorylated; [c] 5´-FAM; [d] 5´-Cy5; A = **dA**[Eln]

2) **Immobilization of β-conglutin**

β-conglutin (2 mg/mL) was immobilized on COOH-functionalized Dynabeads M-270 via carbodiimide coupling as previously described.[3] Unreacted carboxylic groups were blocked with ethanolamine (50 µL, 1M) during a 30 min incubation at 22°C under tilt rotation. The protein-functionalized magnetic beads (3 µL) were washed with PBS buffer (3x 200 µL), blocked with skimmed milk (200 µL, 2% w/v in PBST) for 30 min at 22°C, and washed again with PBS buffer (3x 200 µL). Biotinylated **β-CBA-I** aptamer[3] (50 µL, 100 nM) was incubated for 30 min at 22 °C to confirm the presence of β-conglutin as a qualitative assay. The beads were again washed with PBS (1x 200 µL), followed by addition of SA-poly-HRP (50 µL, 50 ng/mL in PBST) and 30-minute incubation. Following the final wash with PBS (1x 200 µL), TMB (50 µL) was added and the enzymatic reaction stopped by H_2SO_4 (50 µL, 1M). The absorbance at 450nm was also measured for naked beads (no protein immobilized) as a control.

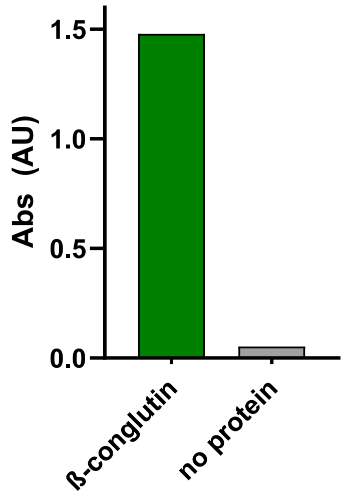

Figure S1. β-conglutin immobilization on COOH-functionalized beads confirmed by qualitative ELAA assay using **β-CBA-I** aptamer.

3) **Library preparation**

3.1 Incorporation of indole-modified dAEInTP by PCR

Incorporation of indole-modified **dA^{EIn}TP** was studied by PCR using different polymerases: KOD XL, Vent(exo-) and TKK18. Reaction mixtures (100 µL)

contained primers (400 nM, Fw, 5´-phosphorylated Rev), ssDNA pool or aptamer candidate template (3 nM), polymerase buffer (1X), dNTP mix (0.2 mM, **dA^{EIn}TP**, dTTP, dCTP, dGTP), and KOD XL (0.075 U/µL) or Vent(exo-) (0.075 U/µL) or TKK18 (0.075 U/µL) DNA polymerase. PCR program included a 5 min initial heating step at 95 °C with a defined number of cycles (as determined by pilot PCR), 30 s denaturation at 95 °C, 30 s annealing (T_a optimized to 50 °C), and elongation (optimized to 30 s) at 72 °C, followed by a final extension for 5 min at 72 °C. Double-stranded PCR products were visualized using agarose gel stained with GelRed and imaged with a UV lamp (λ=254 nm).

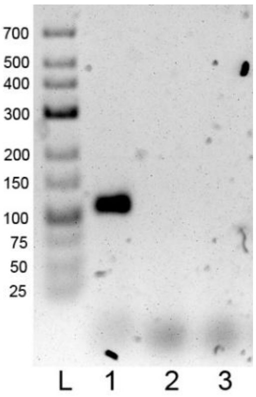

Figure S2. GelRed-stained agarose gel of PCR reactions with **dA^{EIn}TP** using KOD XL (lane 1), Vent(exo-) (lane 2), and TKK18 (lane 3) DNA polymerase.

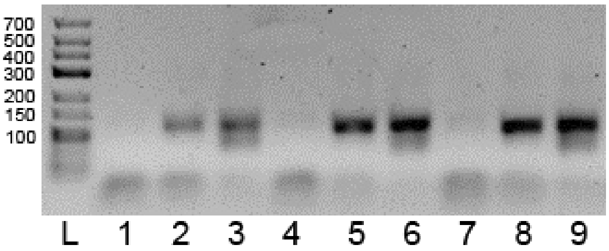

Figure S3. GelRed-stained agarose gel of PCR reactions using KOD XL DNA polymerase with optimization of annealing temperature and number of cycles for synthesis of indole-modified DNA library. T_a = 58°C (lanes 1-3); T_a = 54°C (lanes 4-6); T_a = 50°C (lanes 7-9) under 5 (lanes 1, 4 and 7), 10 (lanes 2, 5 and 8) and 20 (lanes 3, 6 and 9) PCR cycles.

3.2 Generation of single-stranded DNA

Double stranded DNA (dsDNA, 50 µL taken directly from PCR) was incubated with λ-exonuclease (0.4 U/µL) for 120 min at 37°C. The reaction was inactivated by 10 min incubation at 80°C and the product purified using the Oligo Clean &

Concentrator kit following the manufacturer´s protocol. The concentration of single-stranded DNA (ssDNA) was estimated using a GelRed-stained agarose gel by comparing the band intensity with that of a known oligonucleotide concentration using ImageJ program.[4]

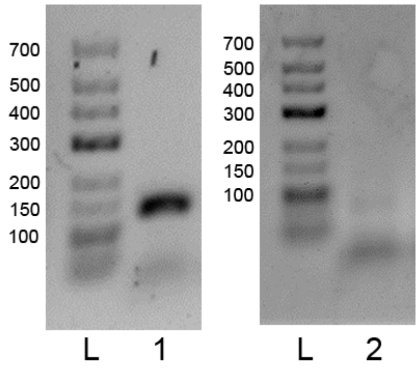

Figure S4. GelRed-stained agarose gels of dsDNA after PCR (lane 1) and ssDNA after digestion with λ-exonuclease (lane 2).

4) Aptamer selection

4.1 SELEX protocol

The library used in SELEX consisted of 50 random positions flanked by primer binding regions (23 and 21 nucleotides) - the reverse primer was phosphorylated, which was required for the generation of single-stranded DNA via λ-exonuclease digestion. Prior to the first round, unmodified ssDNA library (300 pmol) was diluted in PBS binding buffer, denatured at 95 °C for 5 min, and immediately cooled on ice to prevent the formation of secondary structures. The pool was then incubated with β-conglutin-functionalized magnetic beads (5 µL) for 30 min at 22 °C under tilt rotation. Magnetoseparation was subsequently carried out, the supernatant removed, beads washed with PBS (3x 200 µL), resuspended in DNase-free water (20 µL) and used in PCR with **dA^{EIn}TP** to amplify bound DNA using the protocol described in Section 3.1. Modified ssDNA was then generated according to the protocol described in Section 3.2.

Following the first round, a negative selection step (negative selection) was introduced to remove non-specific binders by incubating the ssDNA (100 µL) with naked beads (5 µL of magnetic beads blocked with ethanolamine) for 30 min at 22 °C under tilt rotation. Unbound DNA was then used in the 2nd round of selection (positive selection). Following each round of SELEX, enrichment of binding sequences was evaluated using pilot PCR – pools from positive and negative beads were amplified for 5-13 PCR cycles and analysed on agarose gel to choose the optimal amplification cycles. The pilot PCR showed there was a good evolution in the aptamer pool, with increasing intensity of the bands for positive selection and decreasing intensity for negative selection (Figure S5). Increasing stringency was thus not deemed necessary and the incubation temperature and duration were maintained constant at 30 min at 22 °C throughout the entire selection process.

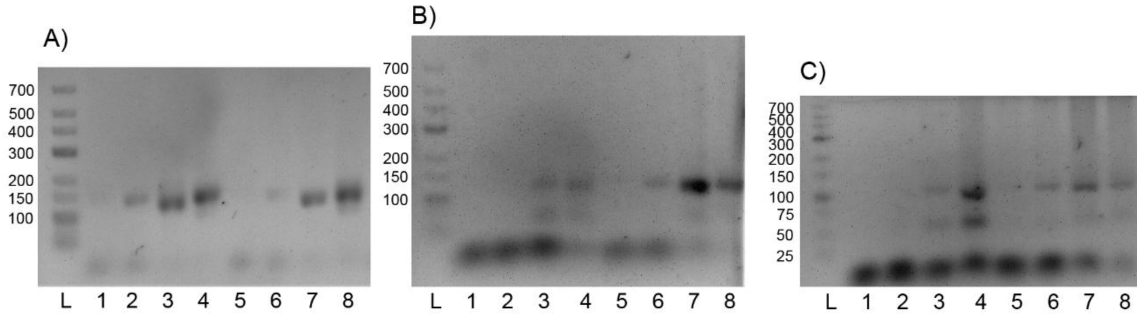

Figure S5. Agarose gels to monitor SELEX evolution with a pilot PCR of round 2 (panel A), 9 (panel B) and 12 (panel C) for negative (lanes 1-4) and positive (lanes 5-8) selection.

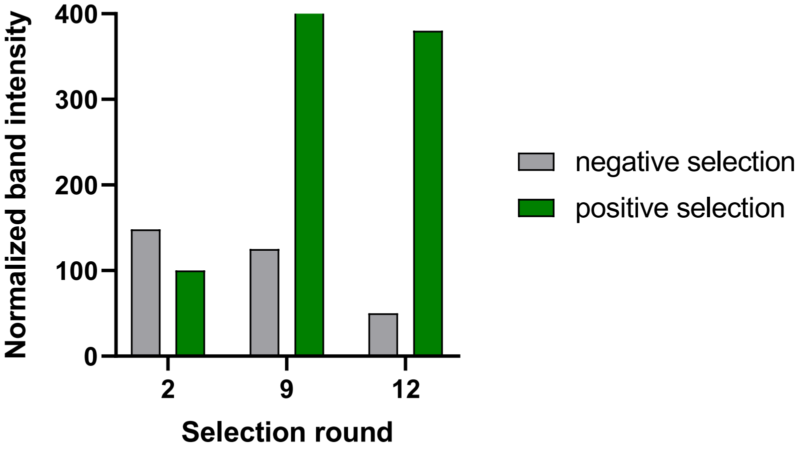

Figure S6. Normalized band intensities for negative and positive beads from 2nd, 9th and 12th selection round.

4.2 Next Generation Sequencing (NGS) and data analysis

Sequences that bound with the naked beads (negative selection) from round 6 and 12, sequences that bound to beads functionalized with β-conglutin (positive selection) from rounds 3, 5, 6, 10, and 12, as well as the initial library that acted as a control, were analysed using NGS. All samples to be sequenced were amplified by PCR using primers to introduce the adapters required for the Ion Torrent NGS platform, as well as individual barcodes incorporated in the forward primers to allow multiplex analysis. The DNA from each of the selected rounds for sequencing (5 µL of beads), or the initial DNA library (1 nM), were amplified in reaction mixtures (50 µL total volume) containing 200 nM of each of NGS_Fw primer and NGS_Rev primer, Dream Taq buffer (1X), dNTPs (0.2 mM) and Dream Taq polymerase (1 U). Double-stranded PCR products were column-purified (DNA Clean & Concentrator kit) and sequenced using Ion Torrent NGS (Centre for Omic Sciences, Eurecat Technology Centre, Reus, Spain).

Raw data in FASTQ format was processed using Cutadapt[5], Seqkit[6], FASTX-Toolkit[7] and FASTAptameR 2.0[8]. A set of eight sequences were chosen for further analysis: (a) the six most abundant sequences (**HB1**, **HB2**, **HB4**, **HB5**, **HB6** and **HB7**) in the 12th round and some of the most representative clusters (1, 2, 3, 4, 6 and 7 respectively, Figure S9), (b) **HB3**, which is among the top 200 sequences and (c) **HB8**, found within the top 100 sequences but not present in the negative selection pool from round 6. The frequencies of these sequences were compared to track their evolution during the selection process (Figure S8). Table S3 summarizes the results of the processed pools, including raw sequences, processed sequences and unique sequences.

Table S3. NGS raw data analysis.

Selection round	Total sequences	Processed sequences	Unique sequences	
**Indole_Lib**	131477	79644	78799	98.94%
**3**	124439	59631	59167	99.22%
**5**	152874	77626	67052	86.37%
**6**	409049	151898	85465	56.26%
**10**	121836	68139	5371	7.88%
**12**	541252	229138	15351	6.70%

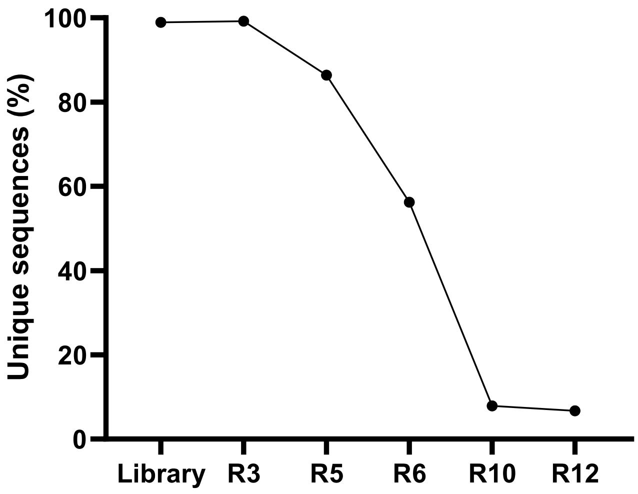

Figure S7. Percentage of unique sequences through SELEX.

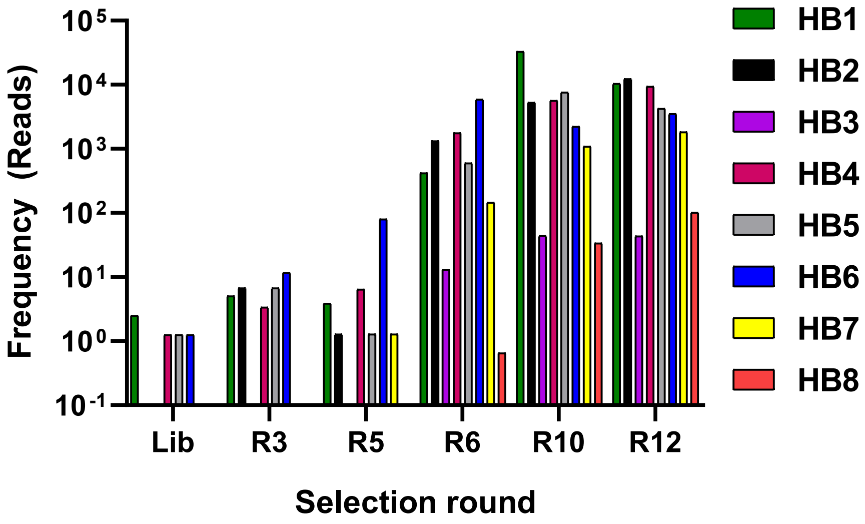

Figure S8. In vitro evolution study of eight aptamer candidates (**HB1-HB8**) from positive selections of different rounds. Frequencies were normalized to 100,000 reads for comparison in between rounds.

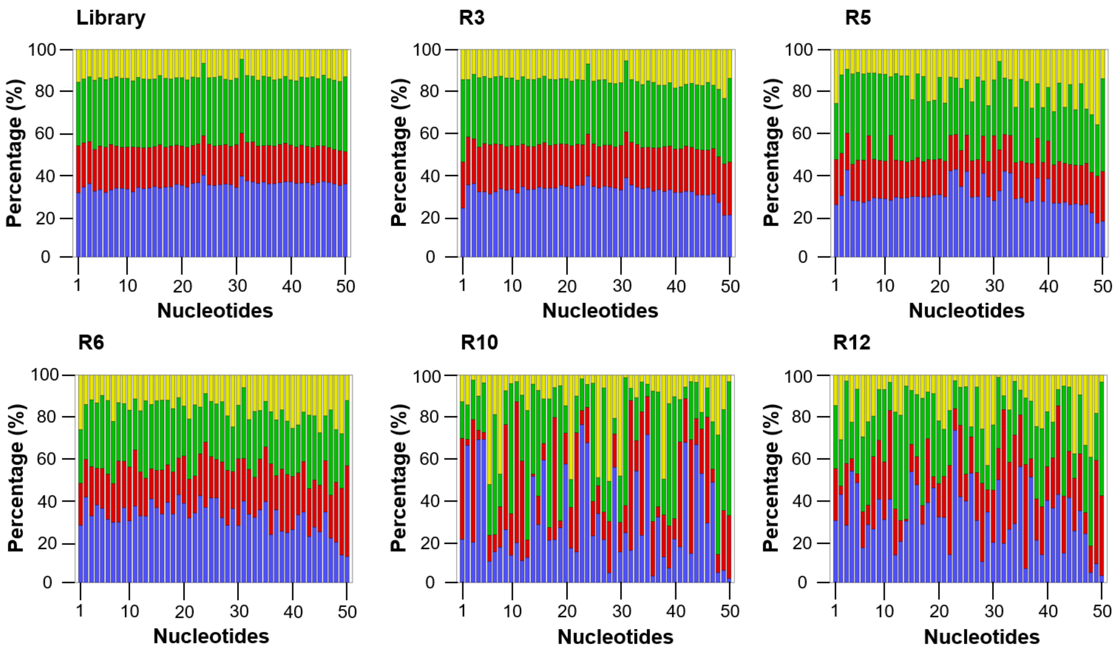

Figure S9. Nucleotide distribution of the randomized region in sequenced pools. Colour legend: yellow = T, green = G, red = C, blue = A.

Table S4. Identified clusters from the 12th selection round.

Cluster	Seed sequence	Sequences	Reads
**1**	ATGTGGACAAAGGGAAGAAGACACGAACTGACCCACAGTAACGGTGTTCG	131	43152
**2**	CACAATGTCGCCGAGAGCGATCAAGTGTATGCACAGGTGGCACAACAGGG	94	32879
**3**	GGAAACGAGCCGTGAGAGATGTAAACCGTAACTGCCAAGATCGCCAGGCC	92	28667
**4**	AACGAGTGAGCGTGCTTCGCCAGACAACCGGCGAAGACCGGAAACATTTC	54	11985
**5**	GAGATTGGAGATGGCACATCAGAGATGGACACCAGGTCCCAAGATCTGTG	58	11920
**6**	TACAAGTCATATGAGGAGAATGACAACCTCATGAACTGCCAAGGTAGCGC	40	9386
**7**	CAAGATCACCGTAGACGCGTCGAGAAGTGTGAGATCCTATGAAATGACCG	54	10779
**8**	TGACGAGATACAATGAAACGCTCCCACAGCGTAAATGCGTATGTGTCTCC	31	4858
**9**	GTGAGATCGGGACGTCGTGAGCAGTAGCACCAGGATGGCTCGCAATCTCC	40	8464
**10**	GTGAGATGCAACAAAGTAGGTGCCAAGATCACCGGAAGATTCGCGAGGAG	39	6964
**11**	CGAAGCCATCCACGTGAAATCGAACCGGCACAACCGGCGGGACGAAGTCG	17	3507
**12**	TACAGATCTGGCGGAGATTAAGCTCCTTAAGATCAGATTTGTGACCGACC	9	2668
**13**	CGGGAGAGATGTCGAAACGGCAGTGACAATCTGGCCAAGATCTCCCAGGA	22	3345
**14**	TTGCGAACGCTGAGAGAAGATCGCAATGCGCACGCCGGTAGTGAGGTGAT	13	2329
**15**	CAATGTGAGATGCAACGAAGCGGGGATCGTCGGCCAAGATCACGATGTAC	16	2071
**16**	TCCACGATGCAGAGATGCCCGGAAATAGGCCAAGATCTGGAACGCACGAC	18	2125
**17**	AAGTGCGTCGAGGGGAGCGAAGATGAAGAGTGATCCGCTTTCGGATGTCC	8	1238
**18**	TATGGATGCCCAAAACGTACGATCAGGTACGAGCCCACTTTGTTTCGTTG	32	3086
**19**	CTCCCATCAGAAAGGGGAAAGTGAGGGTAGGGGAGGCAAAGCGGAAGGTG	8	1073
**20**	AGAGGAAGGTGGGAGGGGCCGAAAAGCTAAGAGATGATTGACAGACCGGA	5	878

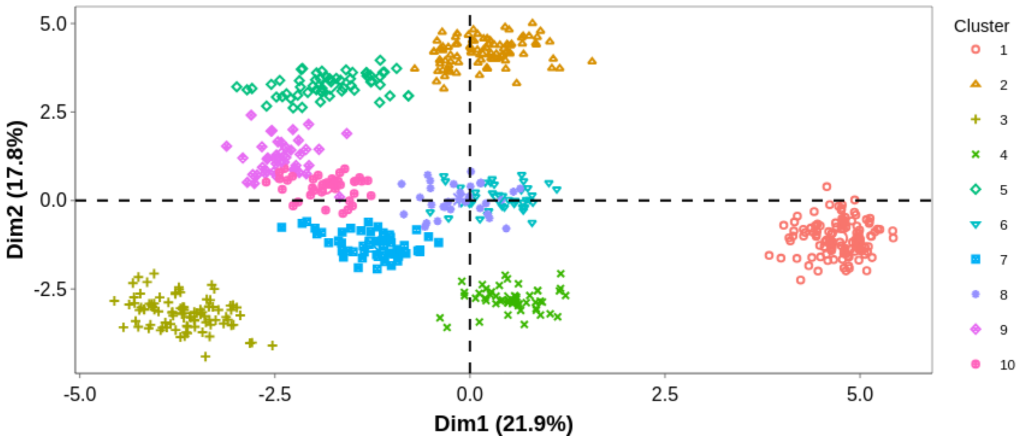

Figure S10. K-mer PCA plot of top 10 clusters obtained from the 12th selection round.

5) Binding affinity and specificity assays

5.1 Aptamer candidate production

All indole-modified candidates for initial ELAA affinity screening were prepared by PCR using the conditions described in Section 3.1, with these differences: 50 µL total reaction volume, 0.6 nM HB candidate template, 5´-biotinylated or 5´-Cy5-labeled Fw primer, 5´-phosphorylated Rev primer, and 18 PCR cycles. Unmodified candidates were prepared using the same protocol using DreamTaq DNA polymerase (0.025 U/µL), 58°C annealing temperature and 15 PCR cycles.

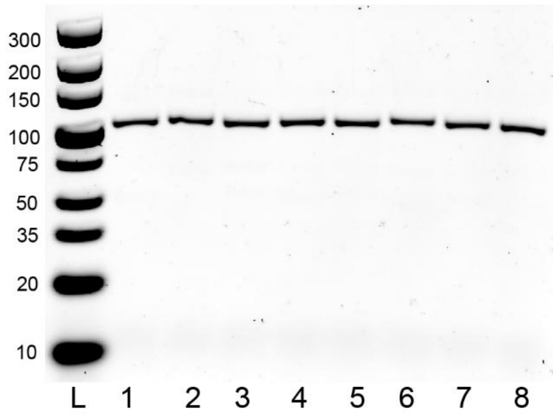

Figure S11. GelRed-stained agarose gel of PCR reactions for all eight HB aptamer candidates.

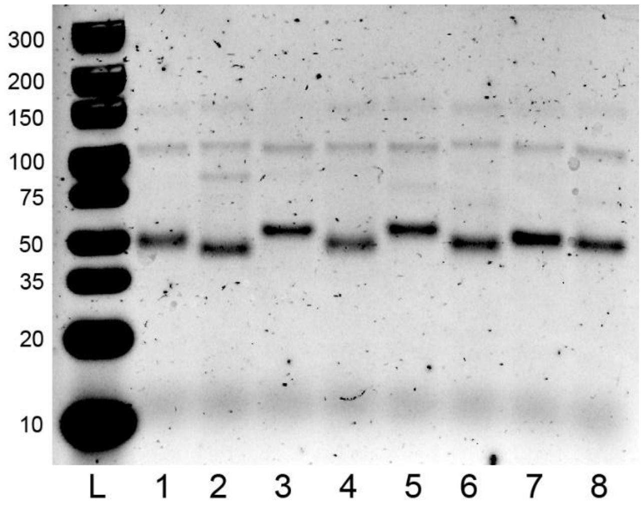

Figure S12. GelRed-stained agarose gel of indole-modified single-stranded HB candidates after λ-exonuclease digestion.

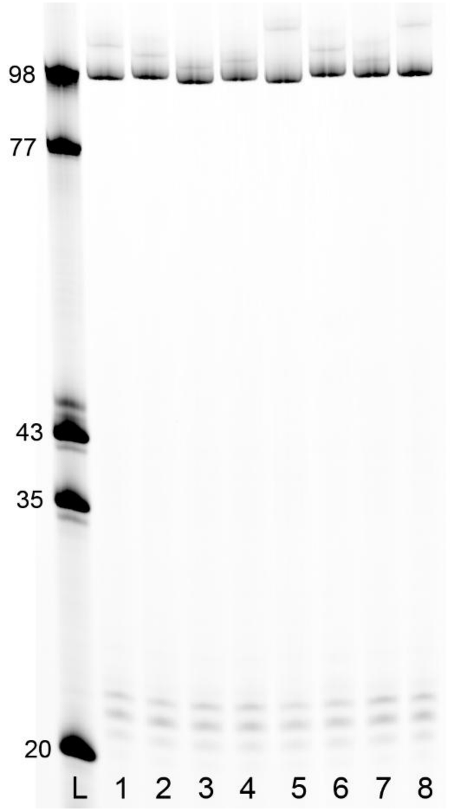

Figure S13. Cy5 scan of PAGE analysis of all eight Cy5-labeled HB candidates.

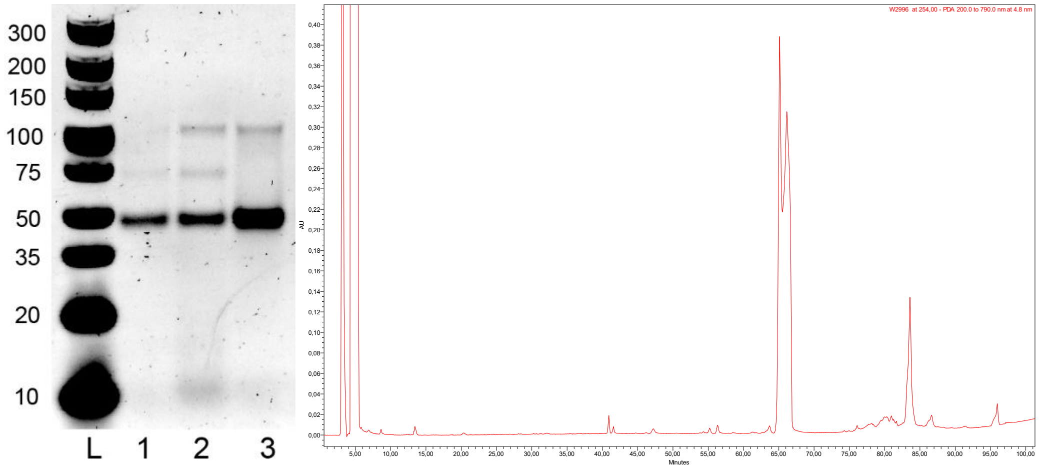

Figure S14. GelRed-stained agarose gel (left panel) of 5´-biotinylated indole-modified single-stranded **HB8** after purification with ssDNA/RNA Clean & Concentrator (lane 1), Oligo Clean & Concentrator (lane 2) or HPLC (lane 3, right panel - product at retention time 83min).

5.2 Enzyme-linked aptamer assay (ELAA)

β-conglutin (20 µg/mL) in carbonate-bicarbonate buffer (50 mM, pH 9.6) was added to the wells of NUNC MaxiSorp plates and incubated at 22 °C for 30 min. The plates were washed with PBST buffer (3x 200 µL) and blocked with skimmed milk (5% w/v, 200 µL in PBST) for 30 min at 22 °C and again washed (1x 200 µL). All 5´-biotinylated aptamer candidates (**HB1-8**, 50 nM) were added to the wells of the β-conglutin-functionalised plates and incubated for 30 min. at 22 °C. Following incubation, the plates were washed with PBST buffer (1x 200 µL), streptavidin poly-HRP (50 µL, 50 ng/mL) was added, and incubated for another 30 min. at 22 °C. After washing with PBST (1x 200 µL), TMB substrate (50 µL) and after 5 minutes the enzymatic reaction was quenched by H_2SO_4 (50 µL, 1M), and absorbance at 450nm was measured. Dissociation constants (KDs) were calculated using the GraphPad Prism software using a specific binding, hill slope, non-linear regression model.

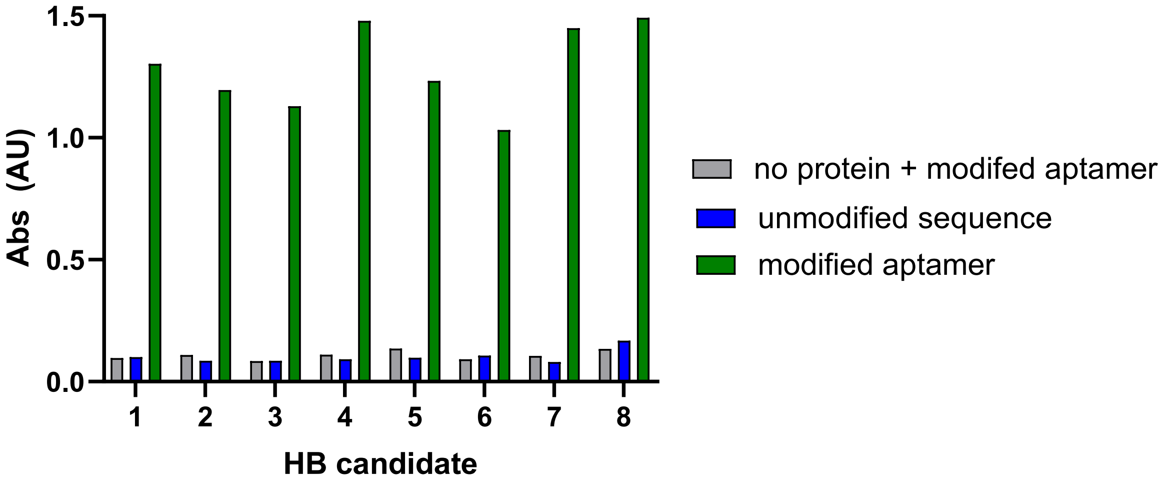

Figure S15. ELAA assay of eight aptamer candidates.

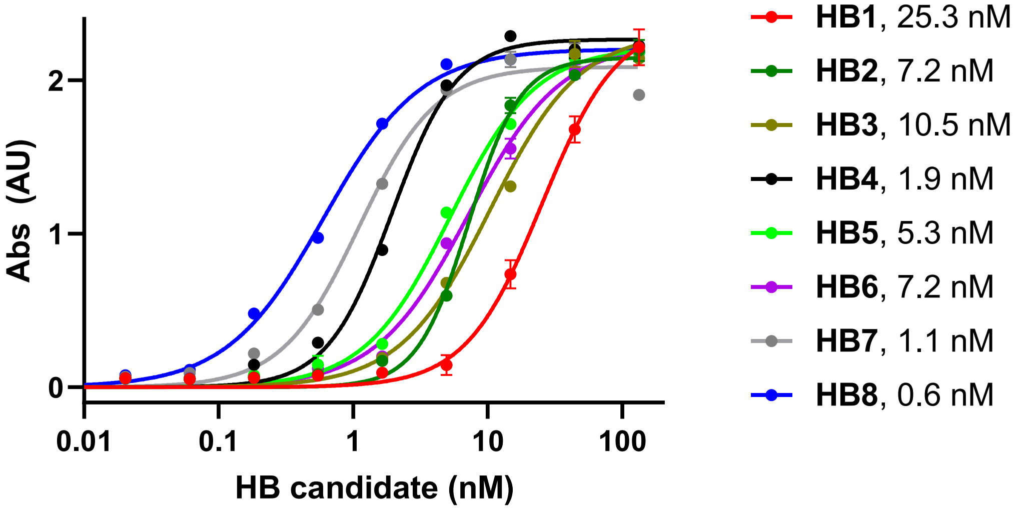

Figure S16. K_D determination of eight aptamer candidates using ELAA assay. Error bars represent the mean ± s.d. from three technical replicates.

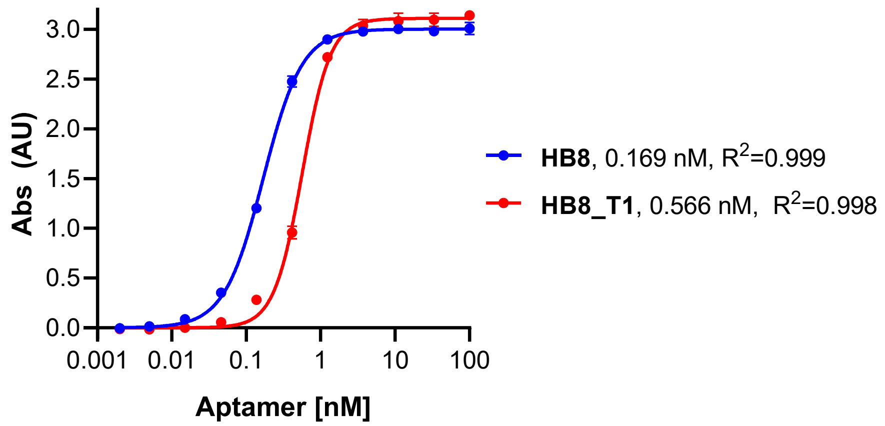

Figure S17. K_D determination of **HB8** and its truncated **HB8_T1** version using ELAA assay. Error bars represent the mean ± s.d. from three technical replicates.

5.2.1 Cross-target selectivity assay

NUNC MaxiSorp plates were functionalized with β-conglutin or Gliadin (20 µg/mL each) diluted in carbonate-bicarbonate buffer (50 mM, pH 9.6) and incubated at 22 °C for 30 min. The plates were washed with PBST (3x 200 µL) and blocked with skimmed milk (200 µL, 5% w/v in PBST) for 30 min at 22 °C. Different concentrations of 5´-biotinylated **HB8** (up to 20 nM) prepared in binding buffer were added to the protein-functionalized wells and incubated at 22 °C for 30 min.. Following thorough washing (1x 200 µL), SA-PolyHRP (50 µL, 50 ng/mL) was added to the plate, followed by TMB substrate (50 µL). The reaction was quenched with H_2SO_4 (50 µL, 1M), and the absorbance was read at 450 nm.

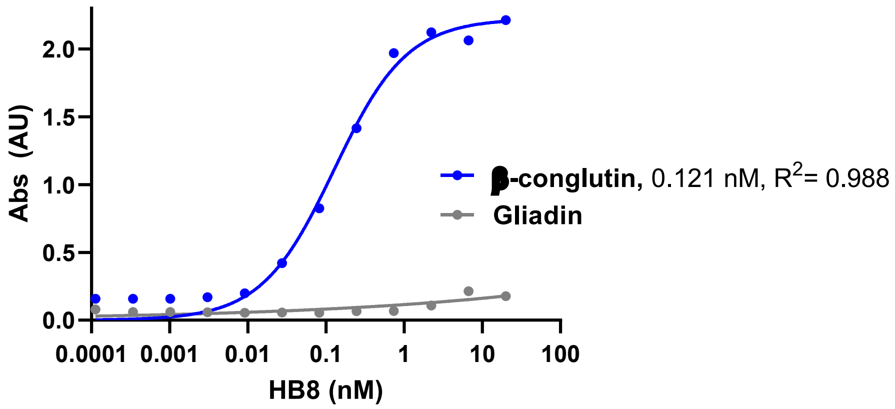

Figure S18. ELAA assay of **HB8** for specificity between β-conglutin gliadin protein.

5.2.2 Competitive assay – LOD determination

5´-biotinylated **HB8** (0.3 nM), **HB8_T1** (0.3 nM) and **β-CBA-II** (0.3 nM) aptamers were pre-incubated with serial dilutions of β-conglutin (0-1000 nM in binding buffer) at 22 °C for 30 min. Subsequently, these complexes were added to the wells functionalised with β-conglutin and incubated at 22 °C for 30 min, and then washed with PBST washing buffer prior to addition of streptavidin-poly-HRP (50 μL, 50 ng/mL in PBST), and a further incubation at 22 °C for 30 min. TMB substrate was then added, and after 5 minutes, the colour development was stopped with sulphuric acid (50 μL, 1M). The absorbance was measured at 450 nm and plotted against the β-conglutin concentration using the GraphPad Prism software. The limit of detection (LOD) was calculated using the sigmoidal dose response (variable slope) model.

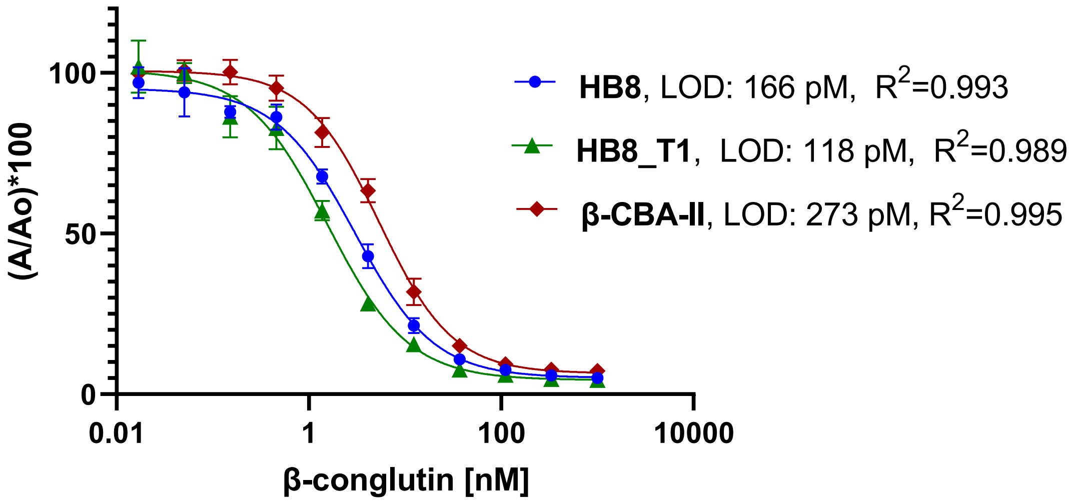

Figure S19. LOD determination by competitive assay using **HB8**, **HB8_T1** and previously published **β-CBA-II** aptamer. Error bars represent the mean ± s.d. from three technical replicates.

5.2.3 Sandwich assay – epitope duality determination

The published 5´-thiolated aptamers β-CBA-I, SGQ and β-CBA-II were immobilized on maleimide-functionalized plates (500 nM) in PBS buffer overnight at 4 °C, and the plate was then blocked with skimmed milk (5% w/v, 200 µL in PBST) at 22 °C for 30 min. β-conglutin (100 µg/mL) was added and the plate again incubated at 22 °C for 30 min. Subsequently, 5´-biotinylated **NSA**, **HB8**, **HB8_T1**, **HB8_SC1** and **HB8_SC2** aptamers (each 100 nM) diluted in binding buffer were introduced and subjected to further incubation at 22 °C for 30 min. As control experiment, Gliadin was used instead of β-conglutin. Subsequently, SA-PolyHRP (50 µL, 50 ng/mL) was added to the plate, followed by TMB substrate addition (50 µL). The enzymatic reaction was quenched with H_2SO_4 (50 µL, 1 M) and the absorbance was read at 450 nm. Results are presented in Figure 2.

5.3 Surface plasmon resonance (SPR)

SPR was performed with the BIAcore 3000 (Biacore Inc.). A CM5 sensor chip activated with a mixture of EDC (30 µL, 400 mM) and NHS (30 µL, 100 mM) was used for the immobilization of β-conglutin (200 µg/mL) at a flow rate of 5 µL/min.

After immobilizing the protein in acetate buffer (10 mM, pH 5), unreacted NHS esters were deactivated using an excess of ethanolamine hydrochloride (1M). The surface was washed with regeneration buffer (2 M NaCl, 50 mM NaOH) to remove any unbound protein at 20 µL/min. **HB8** and **HB8N** (prepared as previously explained) were diluted to the desired concentrations using binding buffer and injected for 6 min at a 5 µL/min flow rate, followed by 3 min stabilization and 7 min dissociation. Binding of the aptamers with the protein was analysed, and the changes produced in the refractive index of the optical signal were expressed as a resonance unit (RU). Any non-specific binding was determined by subtraction of signal from an injection of the aptamer through a cell that had not been modified with the β-conglutin but activated with EDC/NHS and blocked with ethanolamine. Binding affinity was determined using a 1:1 Langmuir binding model using the BioEvaluation software.

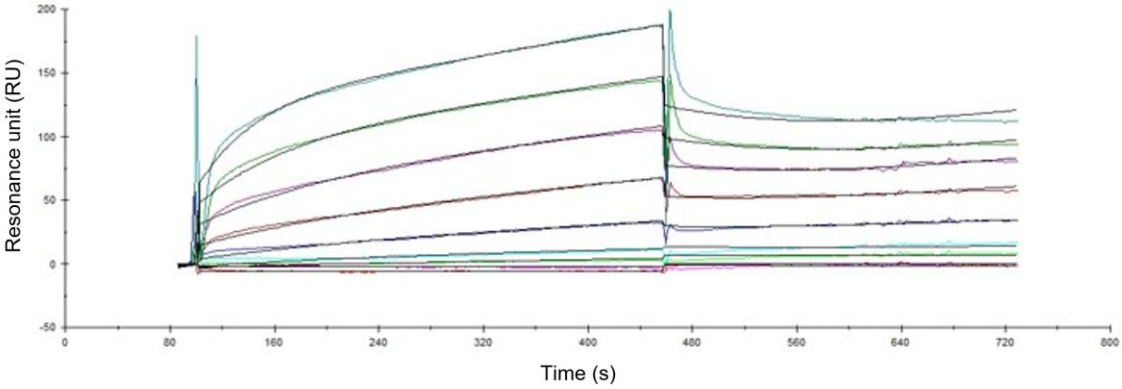

Figure S20. SPR sensorgram of **HB8** aptamer with β-conglutin concentrations (250, 125, 62, 31, 15, 8 4 and 0 nM). K_D = 92 nM.

5.4 Microscale thermophoresis (MST)

5´-Cy5-labeled full-length (**HB8**), its truncated (**HB8_T1**, **HB8_T2**, **HB8_T3**, **HB8_T4**), mutated (**HB8_M1, HB8_M2**) and control (**HB8N**, **HB8_SC1**, **HB8_SC2**) sequences were prepared by PEX as described in Section 6.2. MST experiments were carried out using 20 nM 5´-Cy5-labeled aptamers in binding buffer with 0.1% Pluronic F-127 (for suppression of aggregation and stickiness to glass capillaries) and β-conglutin concentration range: from 1.5 µM for **HB8**, **HB8_T1**, **HB8_T4**, **HB8_M1** and **HB8_M2**; from 3 µM for **HB8_T2** and **HB8_T3**,

and 6 µM for **HB8N**, **HB8_SC1** and **HB8_SC2**. Recorded data was normalized to fraction bound (0 = unbound, 1 = bound) or normalized fluorescence (ΔFnorm) and fitted to a specific binding with hill slope non-linear regression model using GraphPad Prism program.

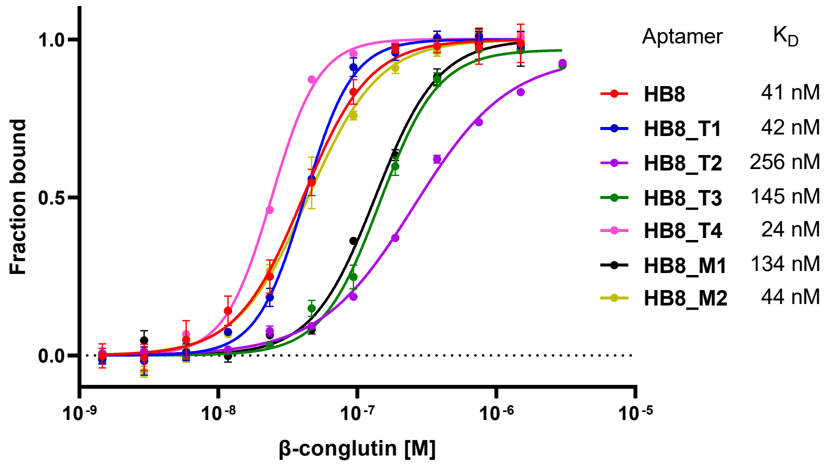

Figure S21. MST measurements of **HB8** aptamer and its truncated and mutated versions with calculated K_D values. Error bars represent the mean ± s.d. from three technical replicates.

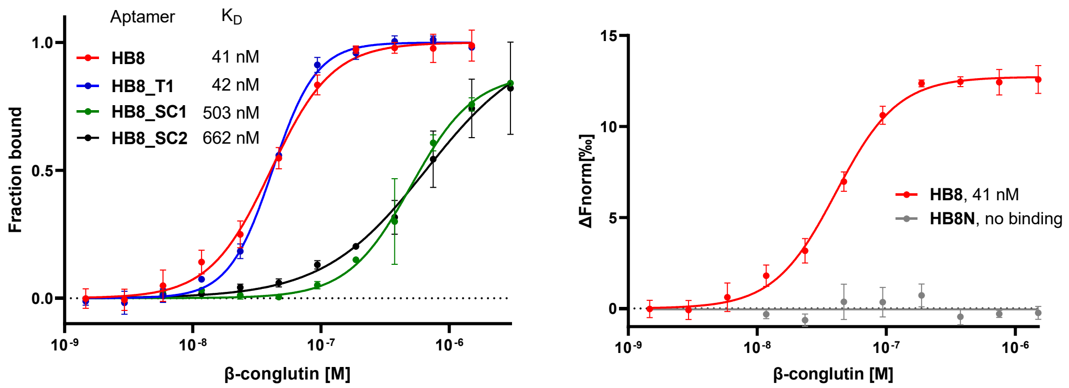

Figure S22. MST measurements of **HB8**, its truncated **HB8_T1** and scrambled (**HB8_SC1** and **HB8_SC2**) sequences (left panel); and **HB8** with its non-modified **HB8N** version (right panel) together with corresponding calculated K_D values. Error bars represent the mean ± s.d. from three technical replicates.

5.5 Biolayer interferometry (BLI)

Binding kinetics of **HB8** and **HB8_T1** were obtained using an Octet RED96 instrument and data were analyzed by GraphPad Prism software assuming 1:1 stoichiometry. Octet AR2G Biosensors (Sartorius, #18-5092) were used following the protocol provided by the supplier. 5´-Biotinylated aptamers were diluted in binding buffer, folded by heating at 95°C for 4 min, and allowed to cool down and temper to room temperature. First, streptavidin (500 nM, sodium acetate buffer, pH 5.5) was immobilized on the biosensors, and aptamers (200 nM, binding buffer) were then captured by the streptavidin. β-conglutin was diluted five-fold from 50 nM with binding buffer and tested for binding. Data was analysed by GraphPad Prism software using an association then dissociation model assuming 1:1 stoichiometry.

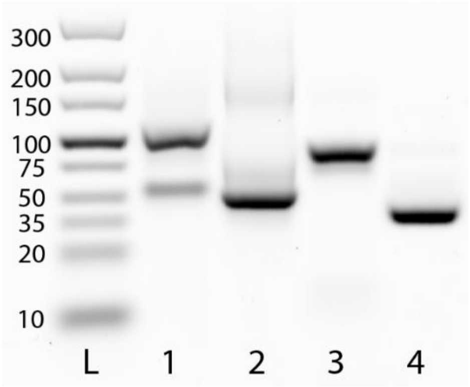

Figure S23. GelRed-stained agarose gel of crude PEX reactions for preparation of **HB8** (lane 1) and **HB8_T1** (lane 3) and their corresponding strand-separated modified strands (lanes 2 and 4, respectively) used in BLI measurements.

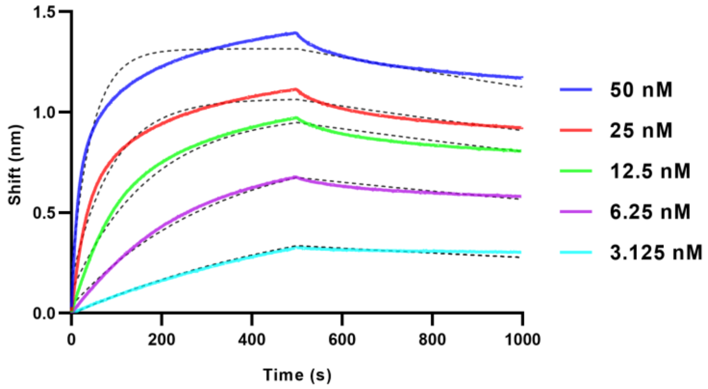

Figure S24. BLI sensogram for **HB8** with calculated K_D = 0.9 nM.

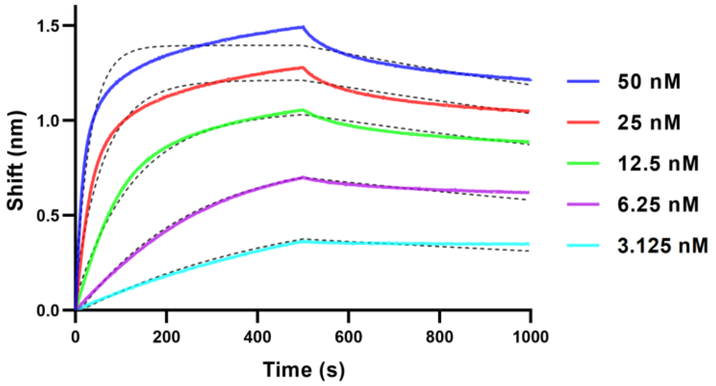

Figure S25. BLI sensogram for **HB8_T1** with calculated K_D =0.6 nM.

6) Aptamer truncation methods

6.1 Synthesis of doubly-labeled primers

Reaction mixture (20 µL) contained FAM-labeled Fw_rG primer (100 µM, 5 µL, Table S1), $dC^{Cy5}TP$ (1 mM, 1 µL), $CoCl_2$ (2.5 mM, 2 µL), Terminal deoxynucleotidyl transferase (TdT, 20 U) and reaction buffer (10X, 2 µL) as supplied by the manufacturer. The reaction mixture was incubated for two hours at 37 °C to achieve a one-nucleotide longer, doubly (5´-FAM and 3´-Cy5)- labeled **Fw_dC^{Cy5}** primer (Figure S25). This primer was then purified by HPLC (Figure S26) and confirmed by ESI (Figure S33).

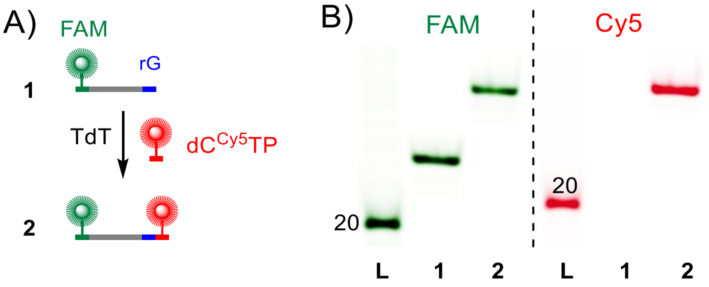

Figure S26. Scheme (A) and FAM and Cy5 scan of PAGE analysis (B) of TdT reaction with Cy5-labeled dCTP. (lane L) 20nt ON as size marker; (lanes 1) FAM-labeled Fw_rG primer; (lanes 2) doubly-labeled (5´-FAM, 3´-Cy5) **Fw_dC^{Cy5}**.

The synthesis described above was multiplied by 10 reactions. The resulting 200 µL was injected for HPLC separation using a C18 column (Waters XBridge Premiere BEH Oligo 4.6x150mm) heated to 60°C with a linear gradient from 0.1

M TEAB in 5% MeCN to 0.1 M TEAB in 20% MeCN in 1 h and to 100% MeCN in 1 h. Buffer pH was adjusted to 7.4 with CO_2 (g). HPLC fraction was freeze-dried.

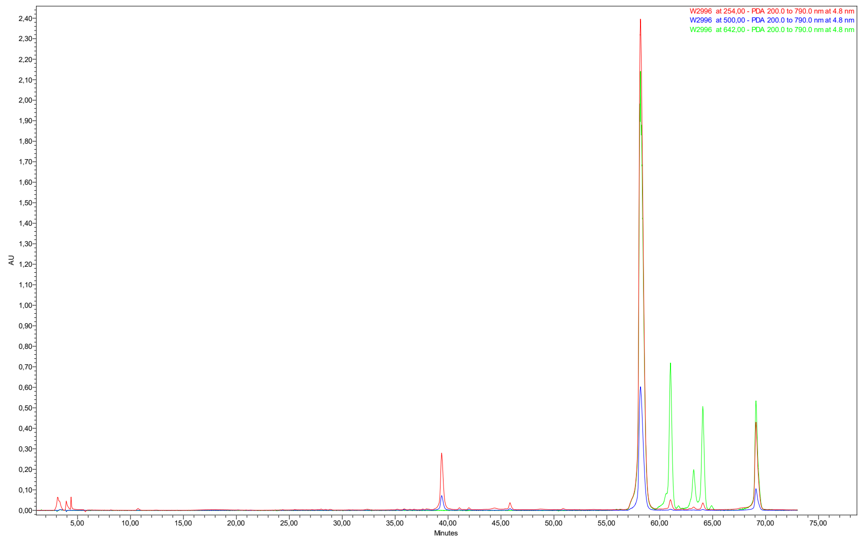

Figure S27. HPLC chromatogram of the purification of the TdT reaction. Crude mixture contained a bit of unreacted Fw_rG primer (39 min), **Fw_dC^{Cy5}** (main peak at 58 min), and primer extended by two dC^{Cy5} nucleotides (69 min).

Reaction mixture (20 µL) contained FAM-labeled Fw_2rG primer (100 µM, 5 µL, Table S1), dC^{Bio}TP (1 mM, 1 µL), $CoCl_2$ (2.5 mM, 2 µL), Terminal deoxynucleotidyl transferase (TdT, 15 U) and reaction buffer (10X, 2 µL) as supplied by the manufacturer. The reaction mixture was incubated for one hour at 37 °C to achieve a one-nucleotide longer, doubly (5´-FAM and 3´-Bio)- labeled **Fw_dC^{Bio}** primer (Figure S27). This primer was then purified by HPLC (Figure S28) and confirmed by ESI (Figure S34).

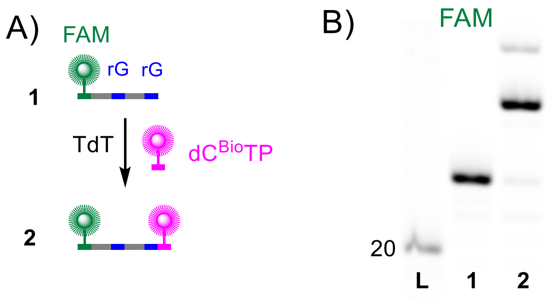

Figure S28. Scheme (A) and FAM scan of PAGE analysis (B) of TdT reaction with Biotin-labeled dCTP. (lane L) 20nt ON as size marker; (lane 1) FAM-labeled Fw_rG primer; (lane 2) doubly-labeled (5´-FAM, 3´-Bio) **Fw_dC^{Bio}**.

The synthesis described above was multiplied by 10 reactions. The resulting 200 µL was injected for HPLC separation using a C18 column (Waters XBridge Premiere BEH Oligo 4.6x150mm) heated to 60°C with a linear gradient from 0.1 M TEAB in 5% MeCN to 0.1 M TEAB in 20% MeCN in 1 h and to 100% MeCN in 1 h. Buffer pH was adjusted to 7.4 with CO_2 (g). HPLC fraction was freeze-dried.

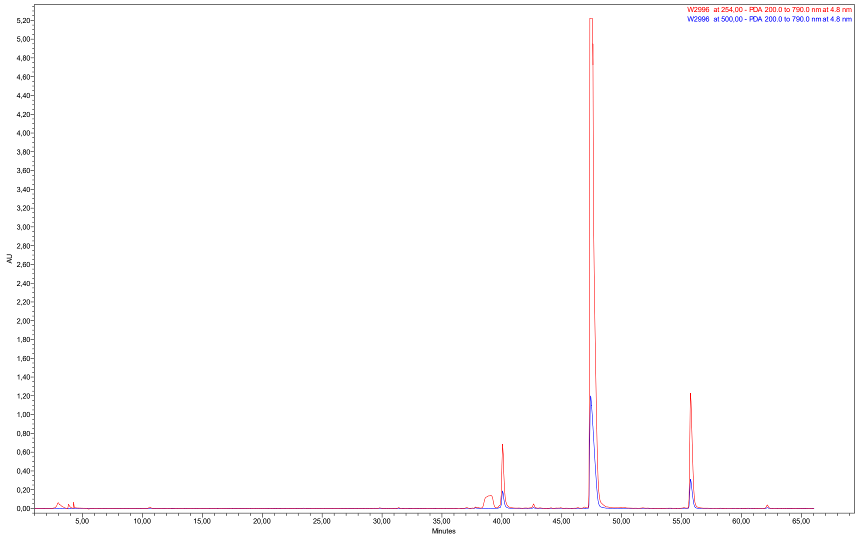

Figure S29. HPLC chromatogram of the purification of the TdT reaction. Crude mixture contained a bit of unreacted Fw_2rG primer (40 min), **Fw_dC^{Bio}** (main peak at 47 min), and primer extended by two dC^{Bio} nucleotides (56 min).

6.2 Primer extension + Magnetoseparation + RNase cleavage

The reaction mixture (20 µL) contained aptamer candidate DNA template (100 µM, 2.5 µL), primer (100 µM, 2 µL), dNTP mix containing **dA^{EIn}TP** (4 mM, 2.5 µL), KOD XL DNA polymerase (2.5 U) and reaction buffer (10X, 2 µL) as supplied by the manufacturer. The reaction mixture was incubated for two hours at 60 °C and then stopped by cooling to 4°C. Crude reaction mixtures were subjected to the following magnetoseparation procedure:

C1 magnetic Dynabeads (150 µL) were used for one PEX (20 µL) reaction. Beads were washed with 1X binding buffer (5 mM Tris-HCl, 0.5 mM EDTA, 1 M NaCl, pH 7.5) (3x 400 µL) and incubated with diluted PEX reaction (20 µL of PEX reaction + 380 µL of water + 400 µL of 2X binding buffer). The mixture was incubated for one hour at room temperature on a Hulamixer. Beads were washed

with binding buffer (3x 400 µL), and 50 µL of 20 mM NaOH was added, vortexed, and incubated for 10 minutes at room temperature using a Hulamixer. Beads were attached to a magnet, and supernatant (~44 µL) containing ssDNA was taken. KOD XL buffer (10X, 5 µL) was added, followed by HCl (0.5 M, 2 µL) neutralization. ssDNA was quantified by Nanodrop at A_{260} or A_{642}.

Samples containing rN within the primer region (50 µL after magnetoseparation) were incubated with RNase AT1 (5 U) for eight hours at room temperature, analysed on dPAGE, and visualised using fluorescence imaging (Figure S29).

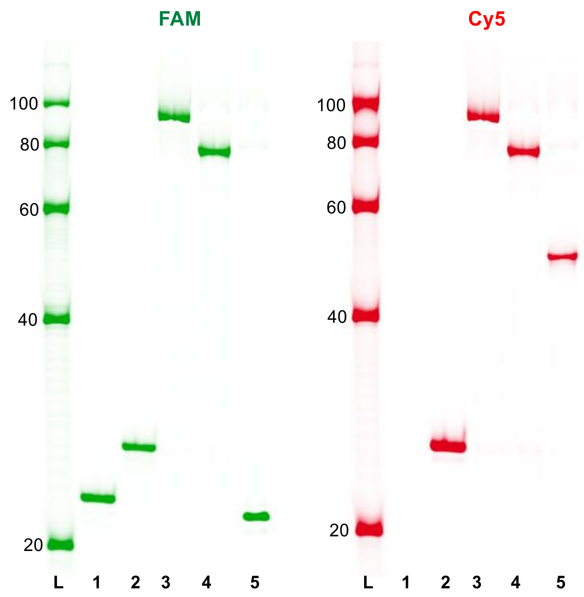

Figure S30. FAM (left) and Cy5 (right) scan of PAGE analysis. Doubly (FAM and Cy5)-labeled ssDNA ladder (lane L); 5´-FAM-labeled Fw_rG primer (lane 1); TdT labeling reaction of Fw_rG primer with $dC^{Cy5}TP$, **Fw_dC^{Cy5}** (lane 2); crude PEX reaction using Fw_dC^{Cy5} primer (lane 3); 5´-biotinylated template removal by magnetoseparation (lane 4); primer removal by Rnase AT1, **HB8_T3** (lane 5).

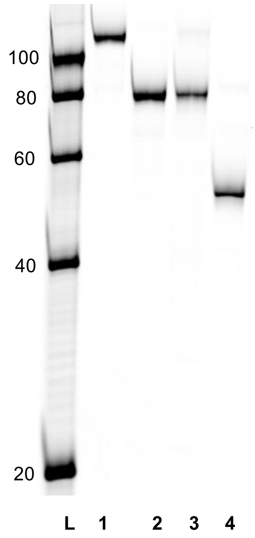

Figure S31. FAM scan of PAGE analysis of PEX reactions. FAM-labeled ssDNA ladder (lane L); **HB8** (lane 1); **HB8_T1** (lane 2); **HB8_T2** (lane 3); **HB8_T3** (lane 4).

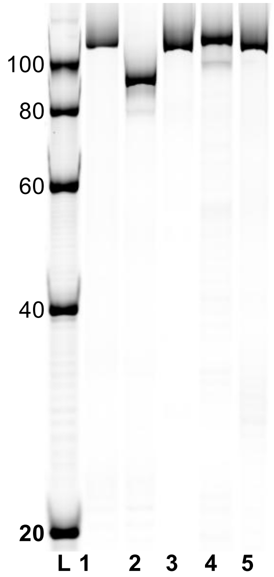

Figure S32. FAM scan of PAGE analysis of PEX reactions. FAM-labeled ssDNA ladder (lane L); **HB8** (lane 1); **HB8_T1** (lane 2); **HB8_M1** (lane 3); **HB8_T4** (lane 4); **HB8_M2** (lane 5).

6.3 Simultaneous λ-exonuclease and RNase cleavage

Reaction mixture (50 µL) contained the crude PEX (20 µL), λ-exonuclease (10 U), RNase AT1 (10 U) and KOD XL buffer (10X, 4 µL). The reaction mixture was incubated at 37°C for 12 hours to achieve complete digestion.

Notes: Buffer supplied with λ-exonuclease inhibits RNase activity, and therefore, the digestion step was performed in KOD XL DNA polymerase buffer. EDTA (1 mM final) can be added after one hour (time necessary for digestion of 5´-phosphorylated template by λ-exonuclease) to avoid further degradation of product by λ-exonuclease activity. A higher EDTA concentration is not recommended due to the inhibition of RNase activity.

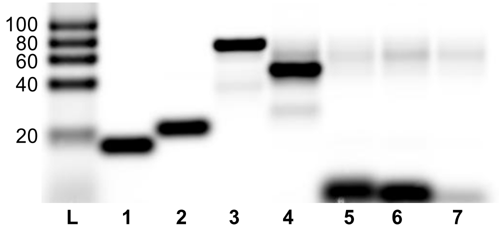

Figure S33. FAM scan of agarose gel. FAM-labeled dsDNA ladder (lane L); 5´-FAM-labeled Fw_2rG primer (lane 1); TdT labeling reaction of Fw_2rG primer with $dC^{Bio}TP$, **Fw_dC^{Bio}** (lane 2); crude PEX reaction using Fw_dC^{Bio} primer (lane 3); 5´-phosphorylated template removal by λ-exonuclease digestion (lane 4); cleavage of rG positions within the primer by RNase AT1 (lane 5); simultaneous digestion of 5´-phosphorylated template by λ-exonuclease and cleavage of rG positions within the primer by RNase AT1 (lane 6); spin column purification for removal of primer fragments (lane 7).

7) MS characterization

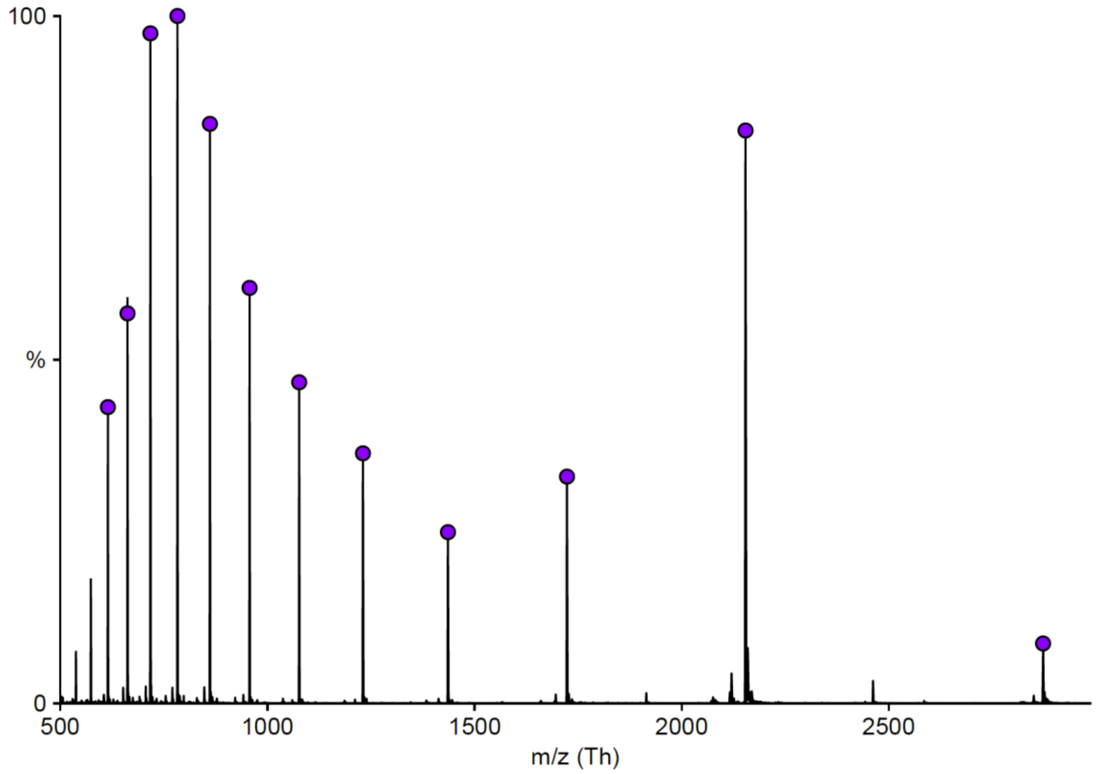

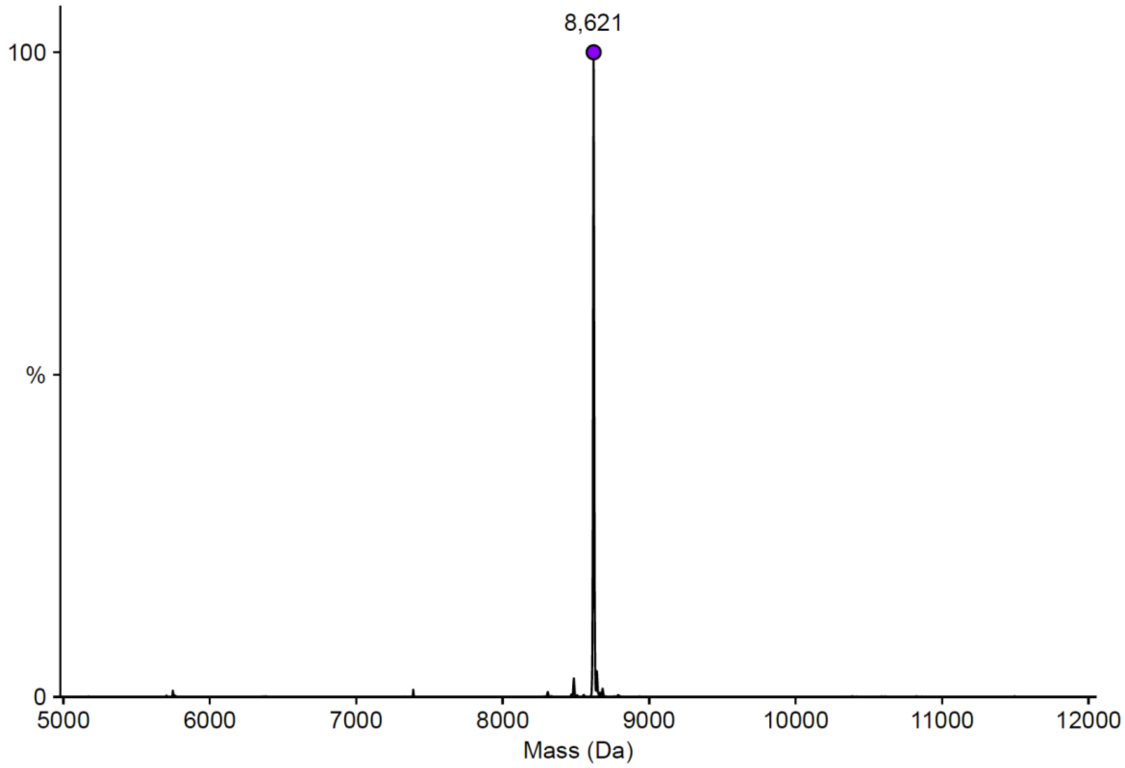

Figure S34. Raw and deconvoluted MS spectrum of **Fw_dC^{Cy5}**, calculated mass: 8621 Da, found mass: 8621 Da.

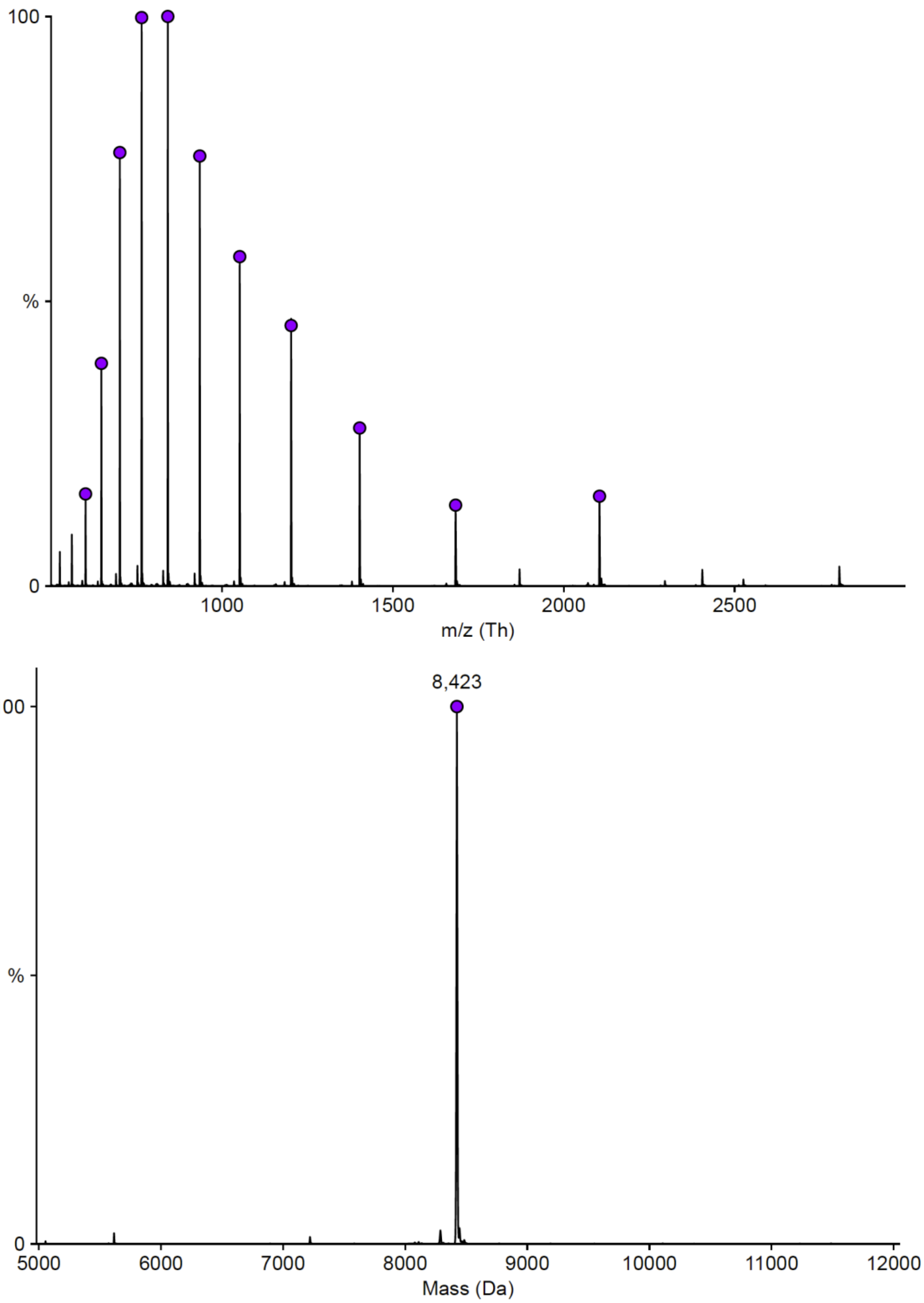

Figure S35. Raw and deconvoluted MS spectrum of **Fw_dC^{Bio}**, calculated mass: 8422 Da, found mass: 8423 Da.

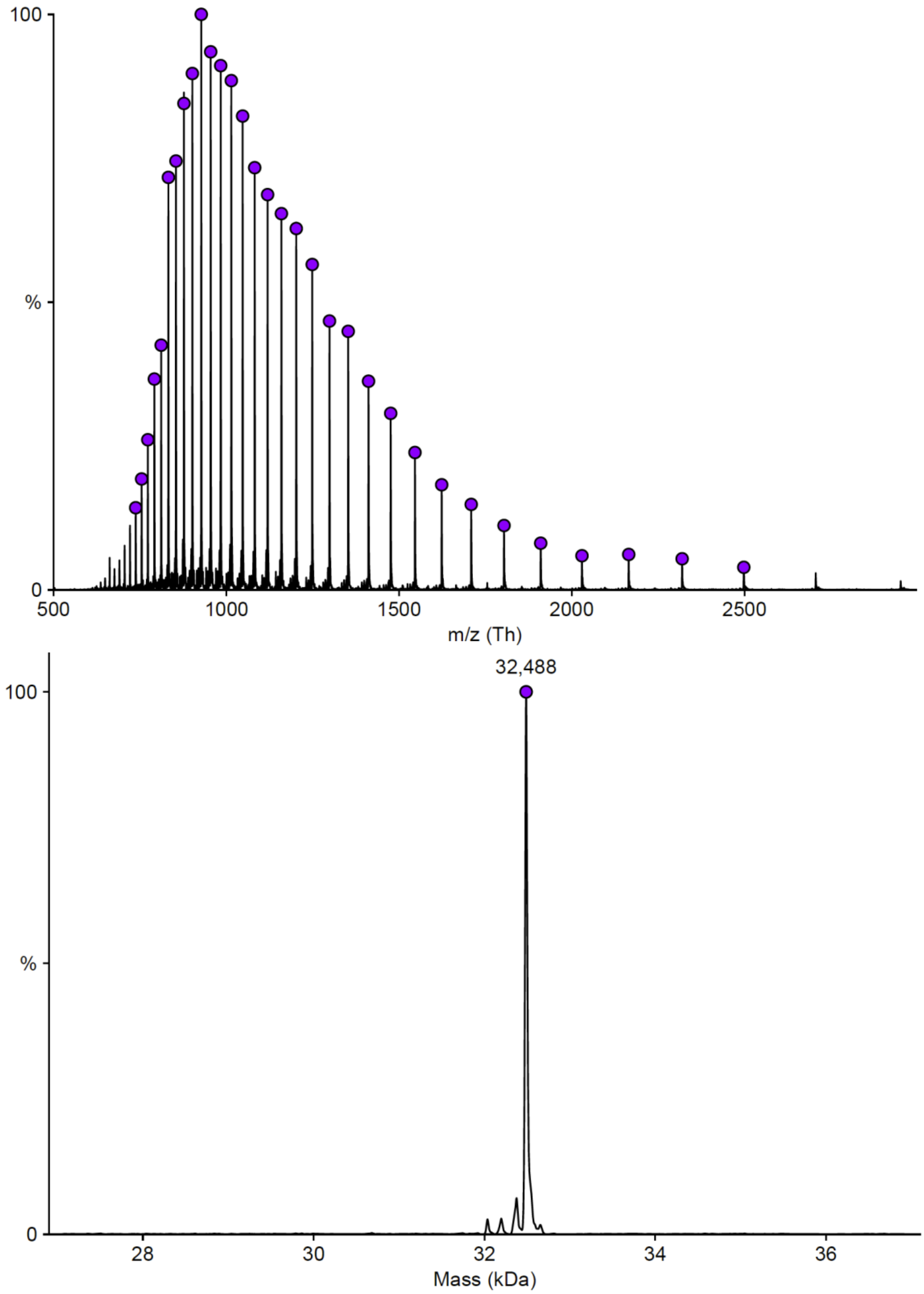

Figure S36. Raw and deconvoluted MS spectrum of 5´-Cy5-**HB8**, calculated mass: 32503 Da, found mass: 32488 Da.

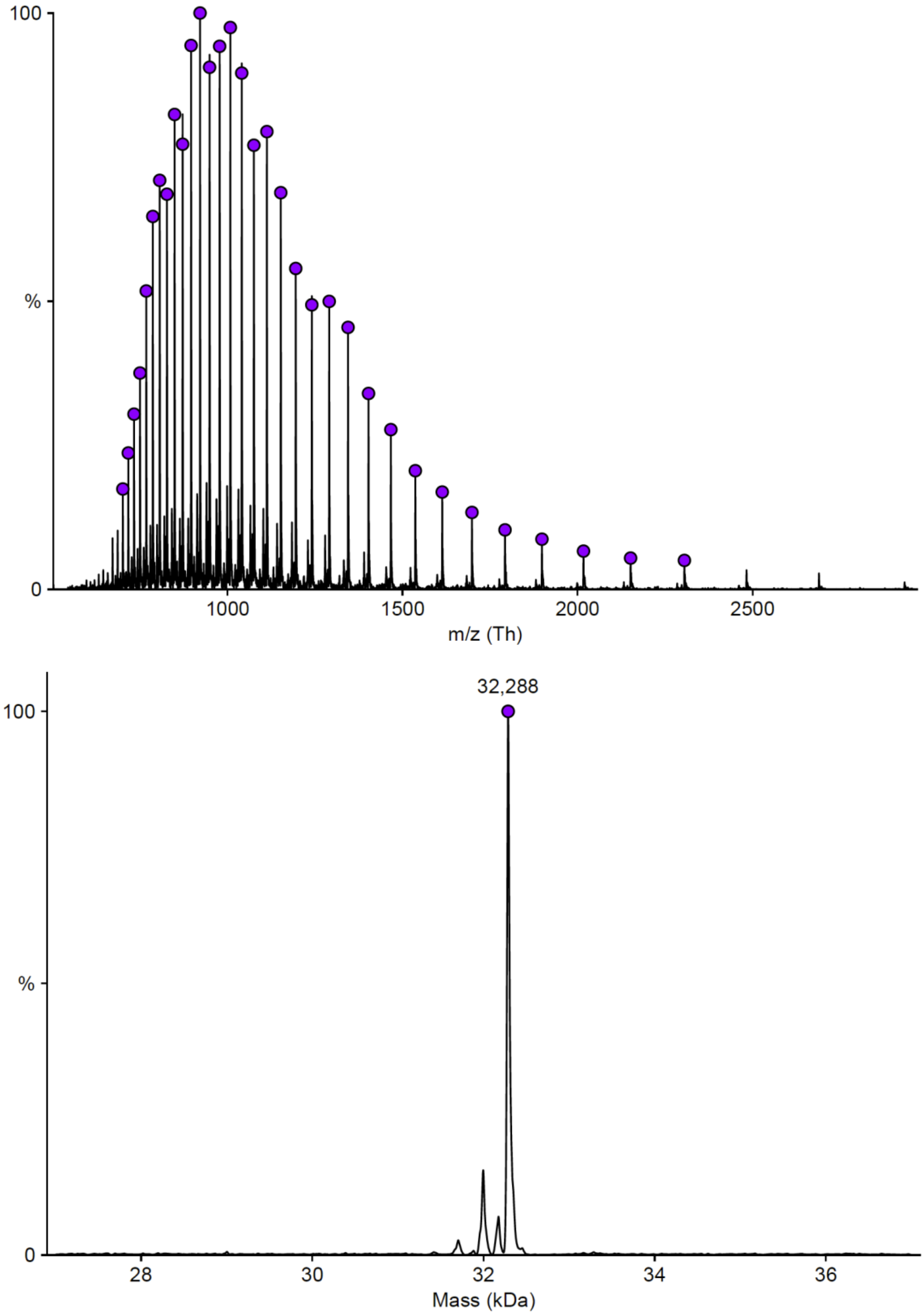

Figure S37. Raw and deconvoluted MS spectrum of 5´-Biotin-**HB8**, calculated mass: 32296 Da, found mass: 32288 Da.

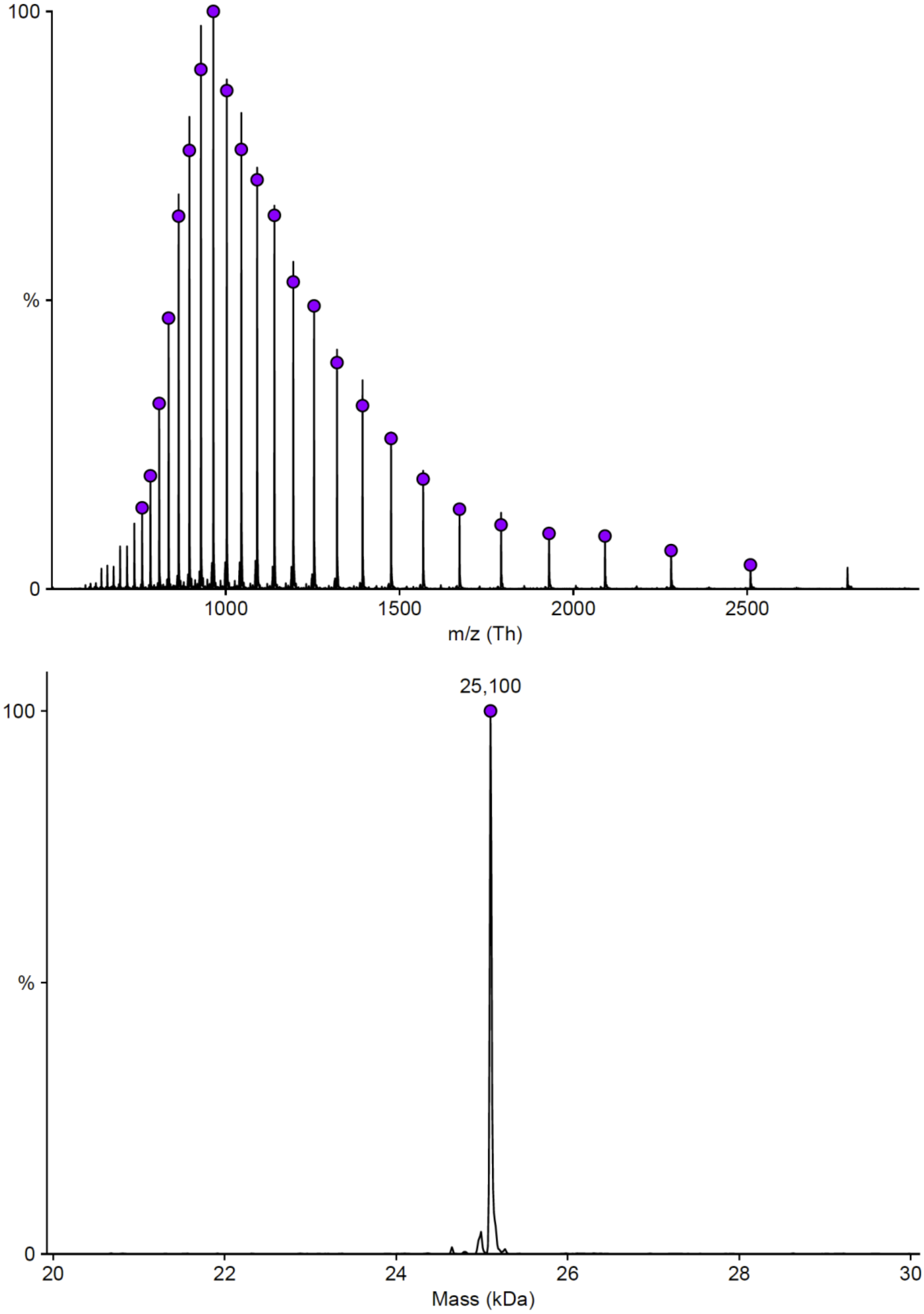

Figure S38. Raw and deconvoluted MS spectrum of 5´-Cy5-**HB8_T1**, calculated mass: 25115 Da, found mass: 25100 Da.

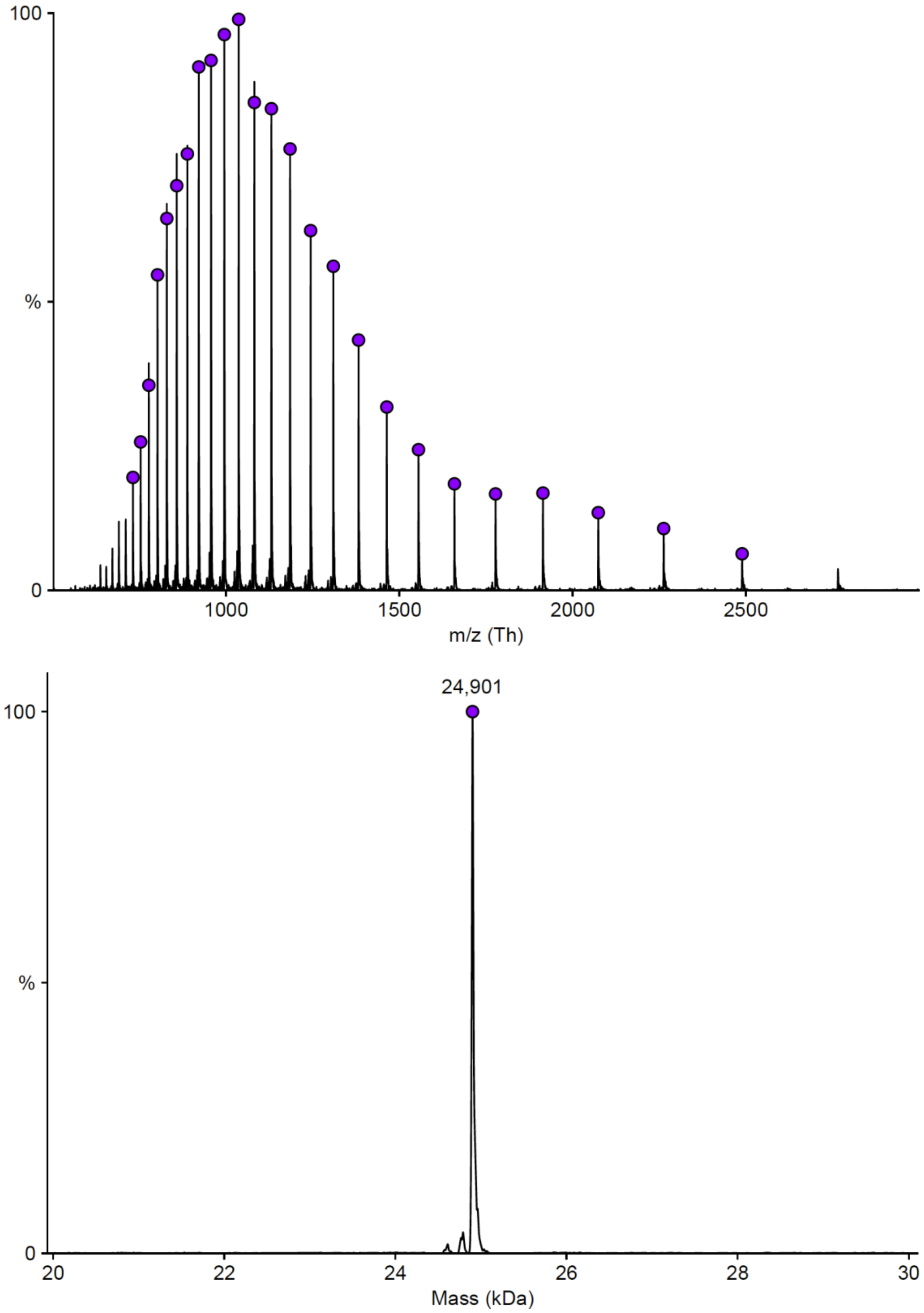

Figure S39. Raw and deconvoluted MS spectrum of 5´-Biotin-**HB8_T1**, calculated mass: 24908 Da, found mass: 24901 Da.

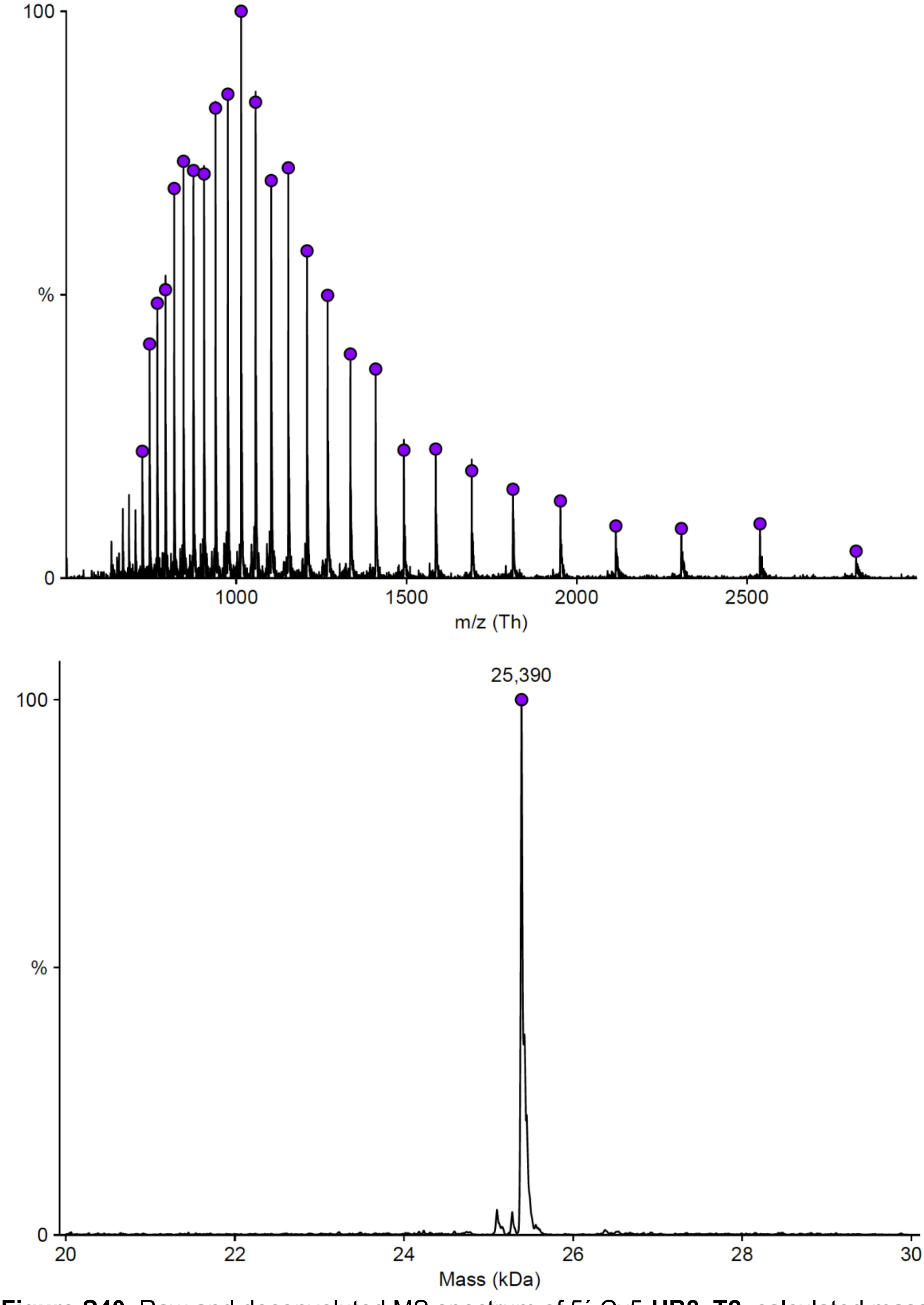

Figure S40. Raw and deconvoluted MS spectrum of 5´-Cy5-**HB8_T2**, calculated mass: 25400 Da, found mass: 25390 Da.

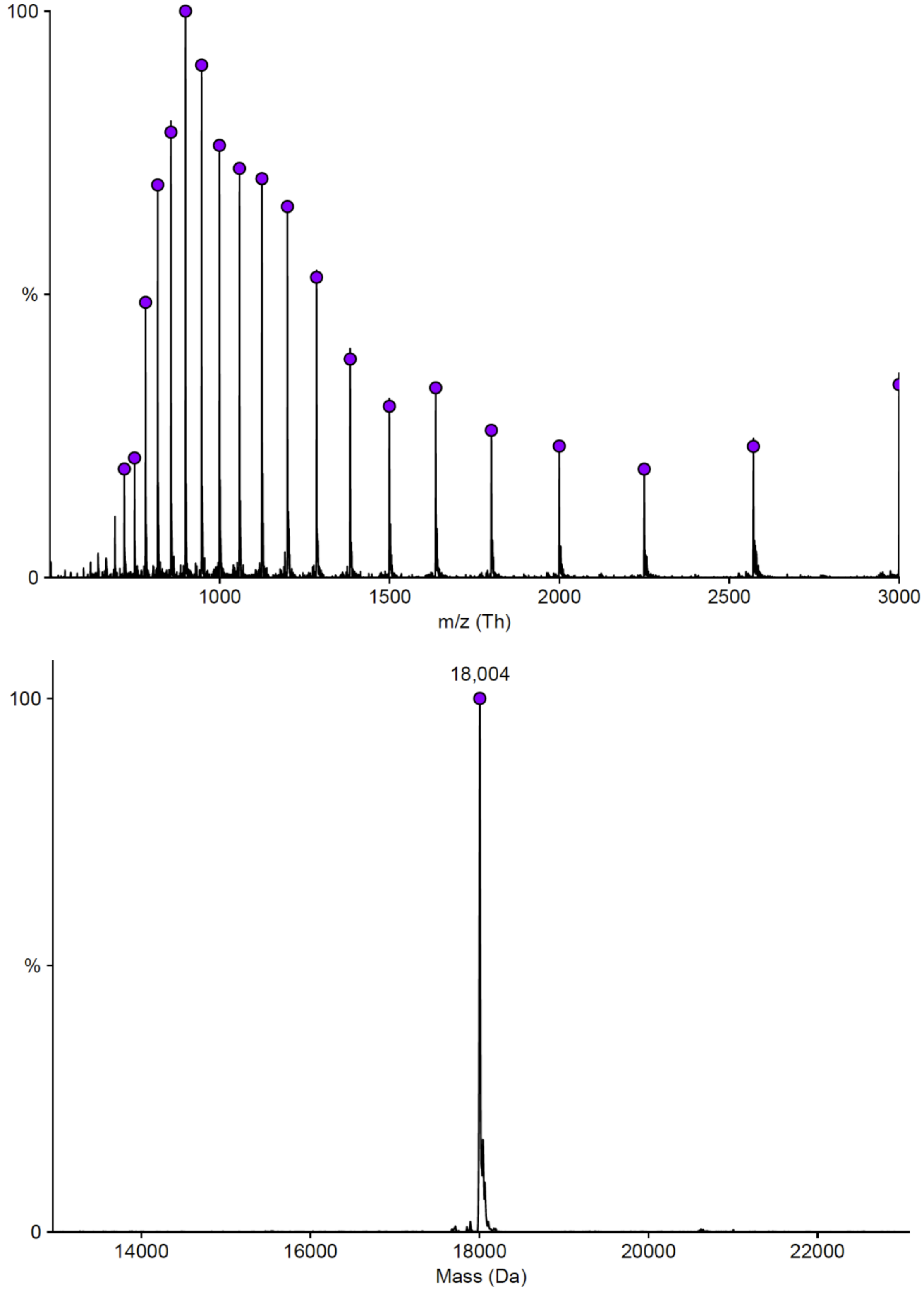

Figure S41. Raw and deconvoluted MS spectrum of 5´-Cy5-**HB8_T3**, calculated mass: 18011 Da, found mass: 18004 Da.

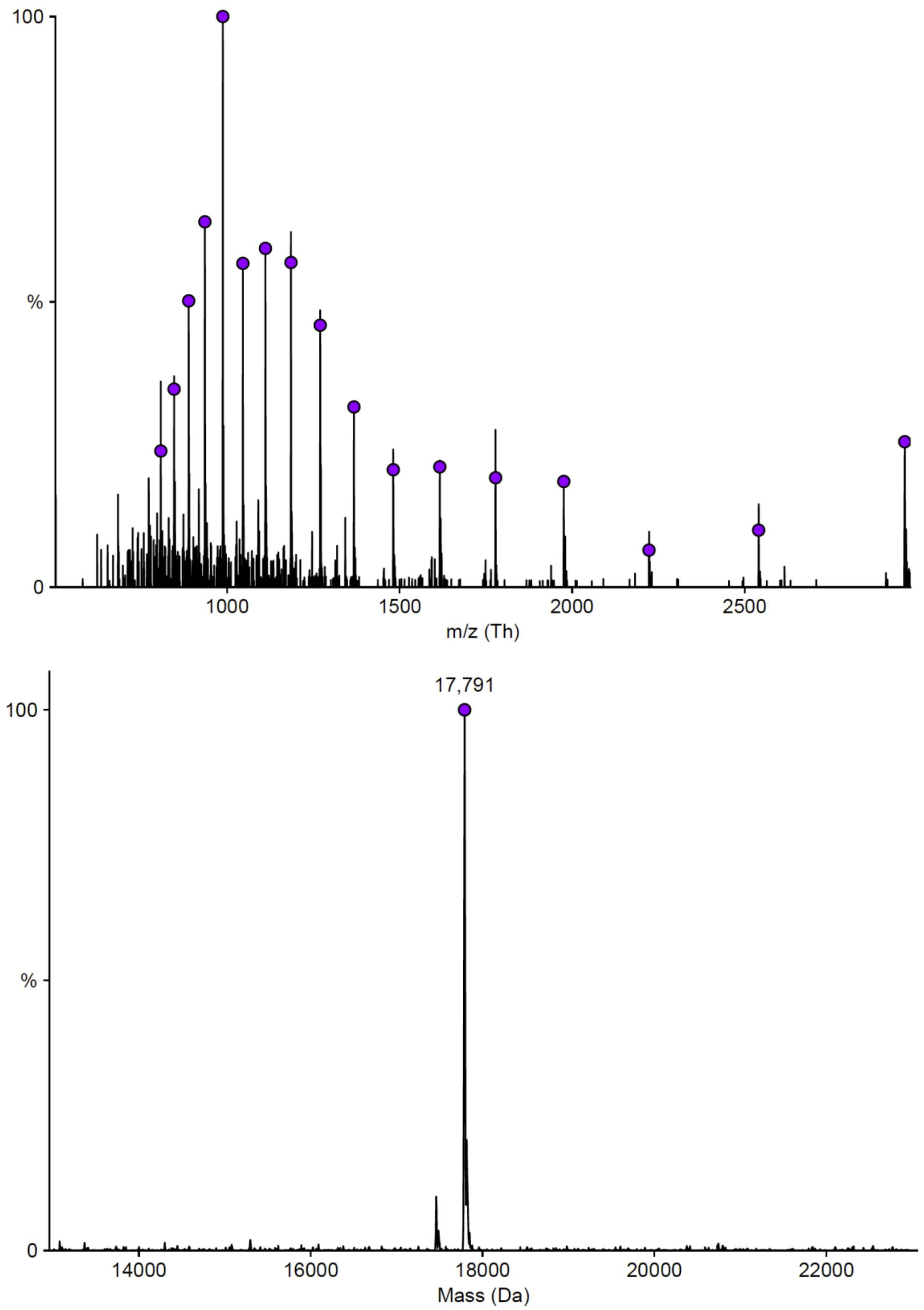

Figure S42. Raw and deconvoluted MS spectrum of 5´-Biotin-**HB8_T3**, calculated mass: 17796 Da, found mass: 17791 Da.

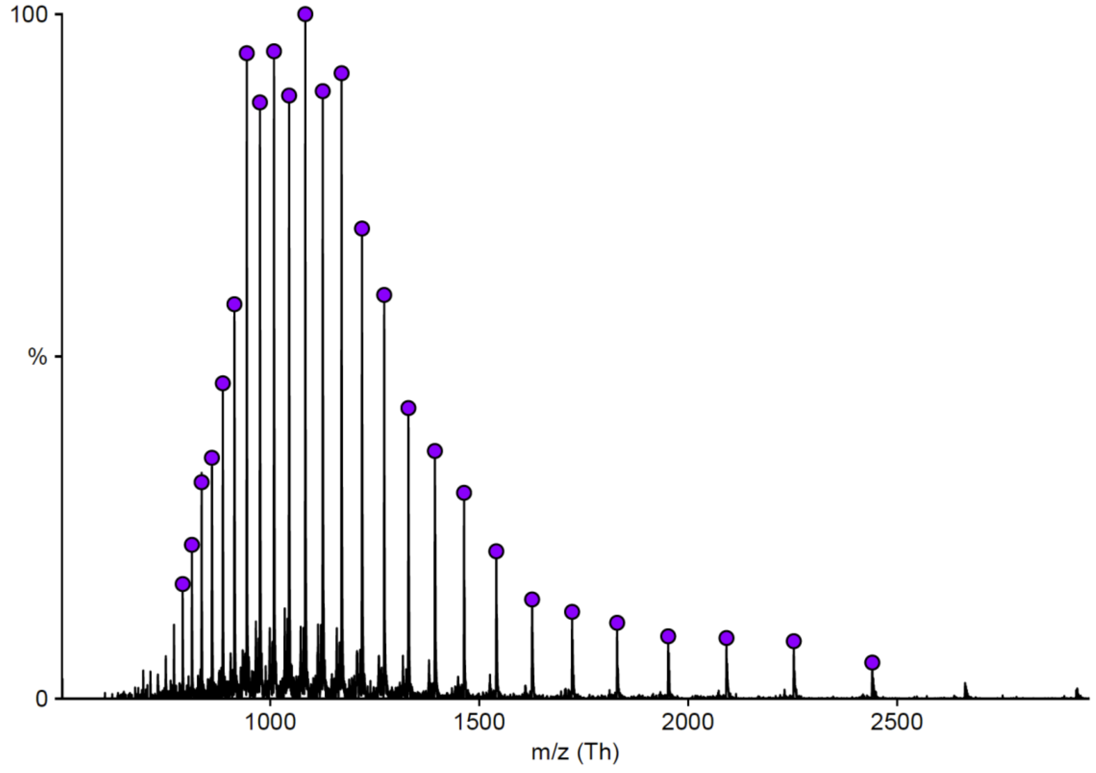

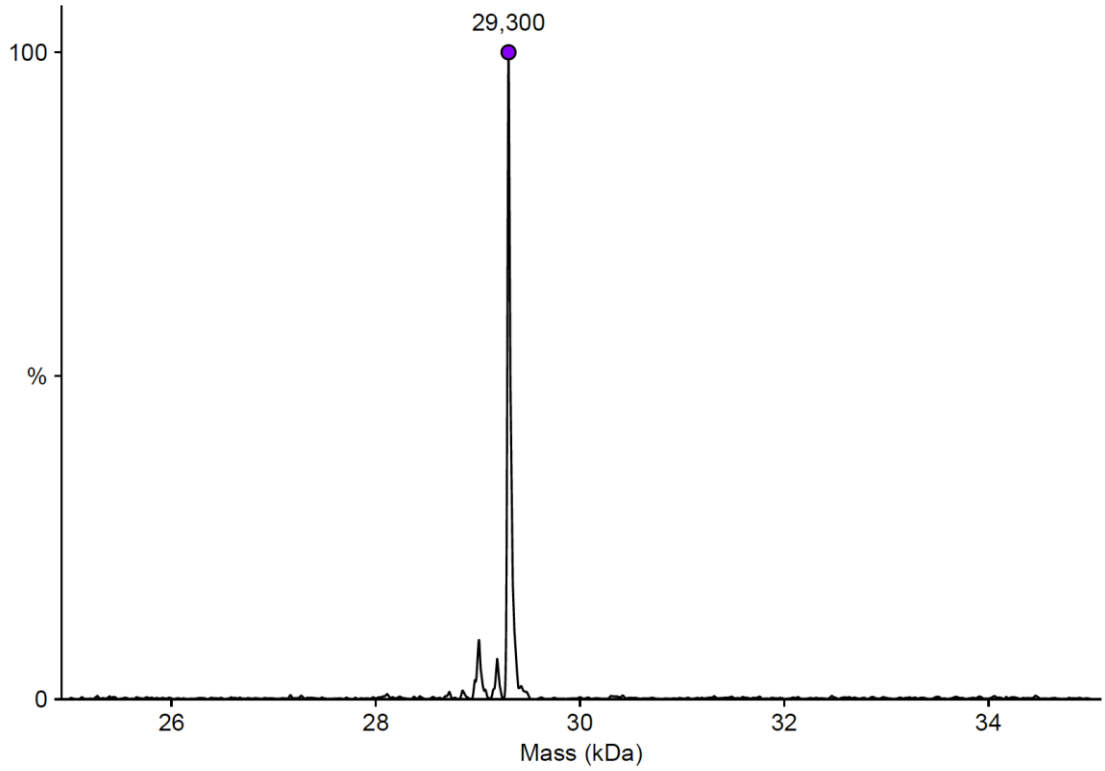

Figure S43. Raw and deconvoluted MS spectrum of 5´-Cy5-**HB8_T4**, calculated mass: 29309 Da, found mass: 29300 Da.

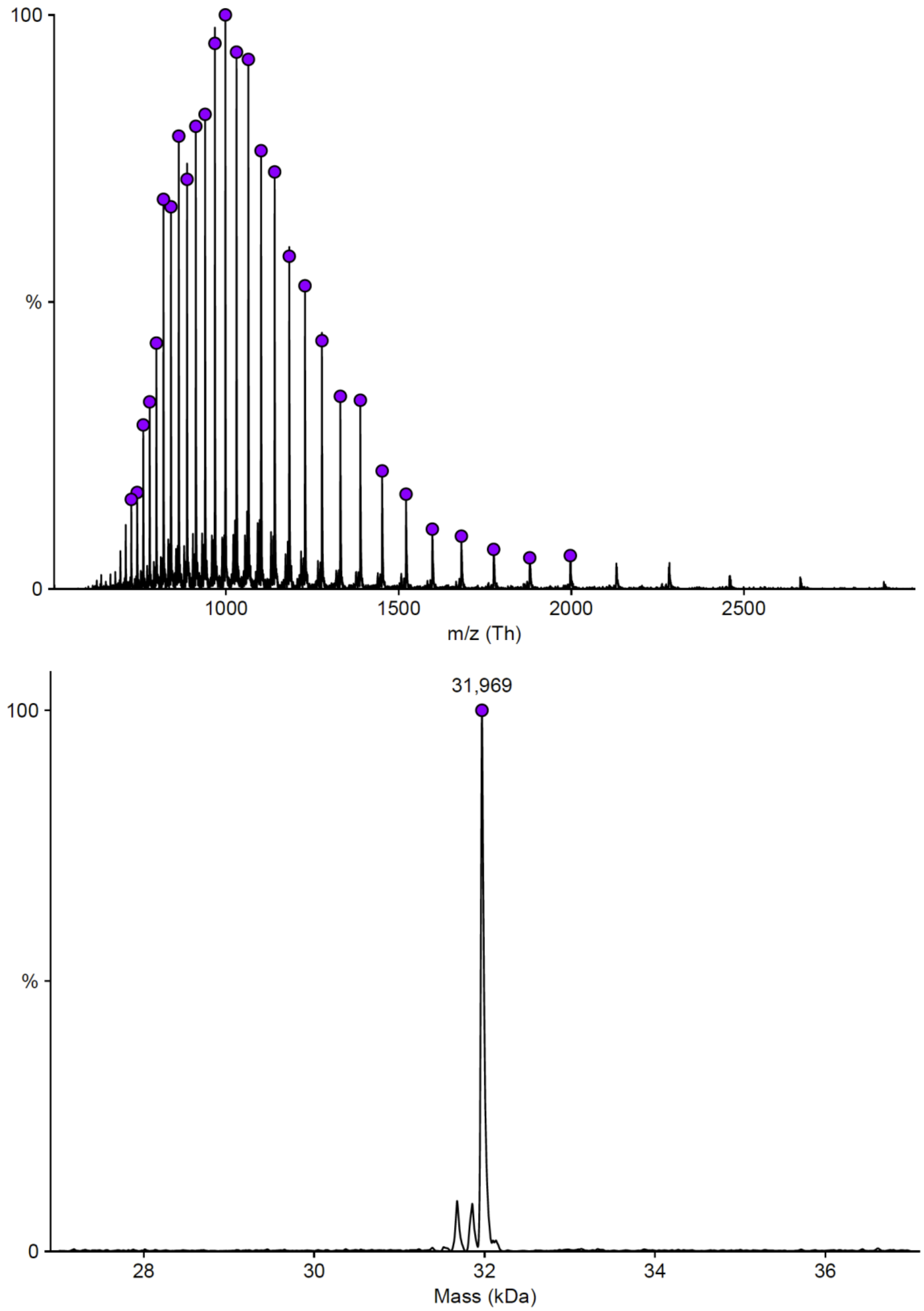

Figure S44. Raw and deconvoluted MS spectrum of 5´-Cy5-**HB8_M1**, calculated mass: 31977 Da, found mass: 31969 Da.

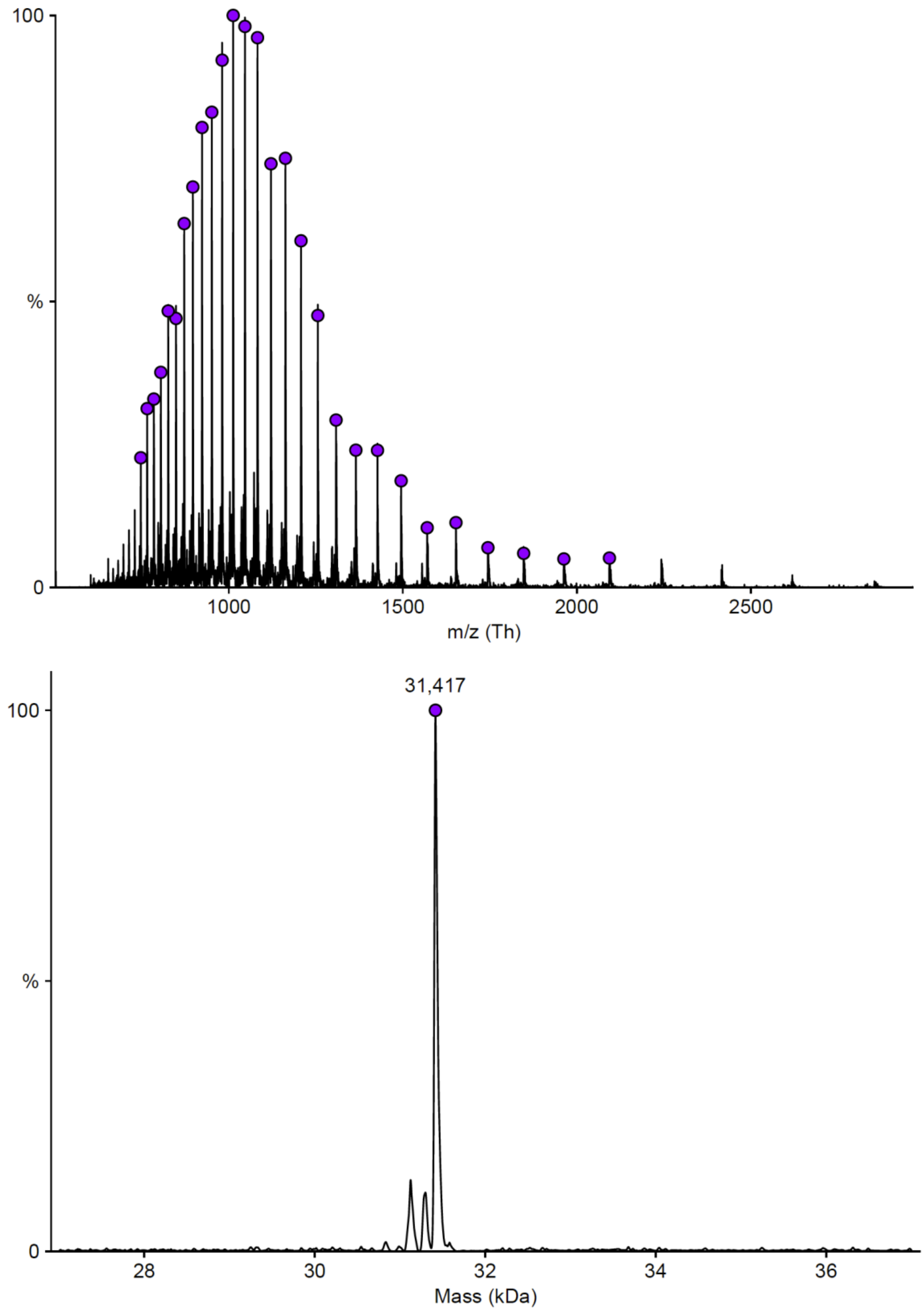

Figure S45. Raw and deconvoluted MS spectrum of 5´-Cy5-**HB8_M2**, calculated mass: 31425 Da, found mass: 31417 Da.

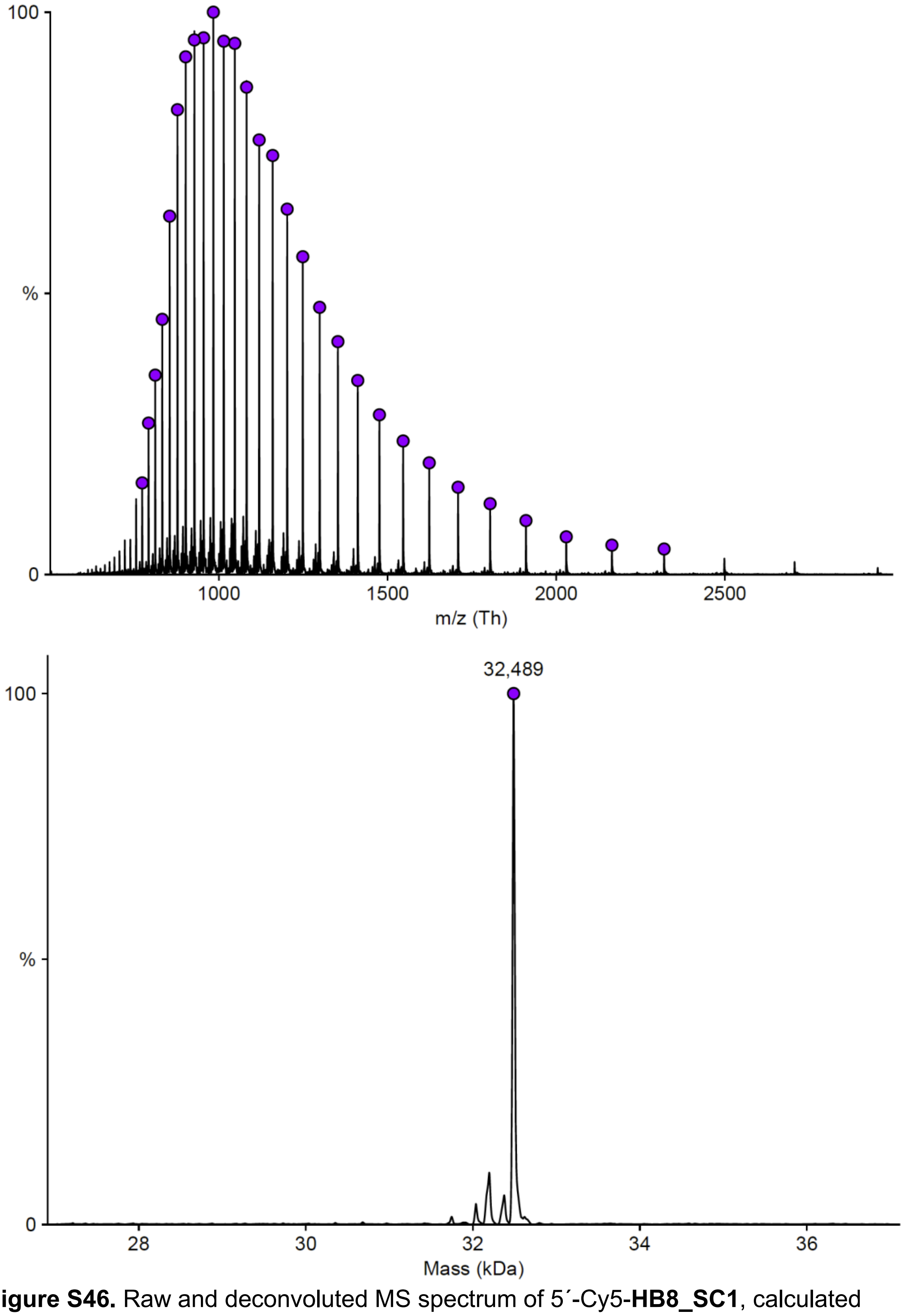

Figure S46. Raw and deconvoluted MS spectrum of 5´-Cy5-**HB8_SC1**, calculated mass: 32503 Da, found mass: 32489 Da.

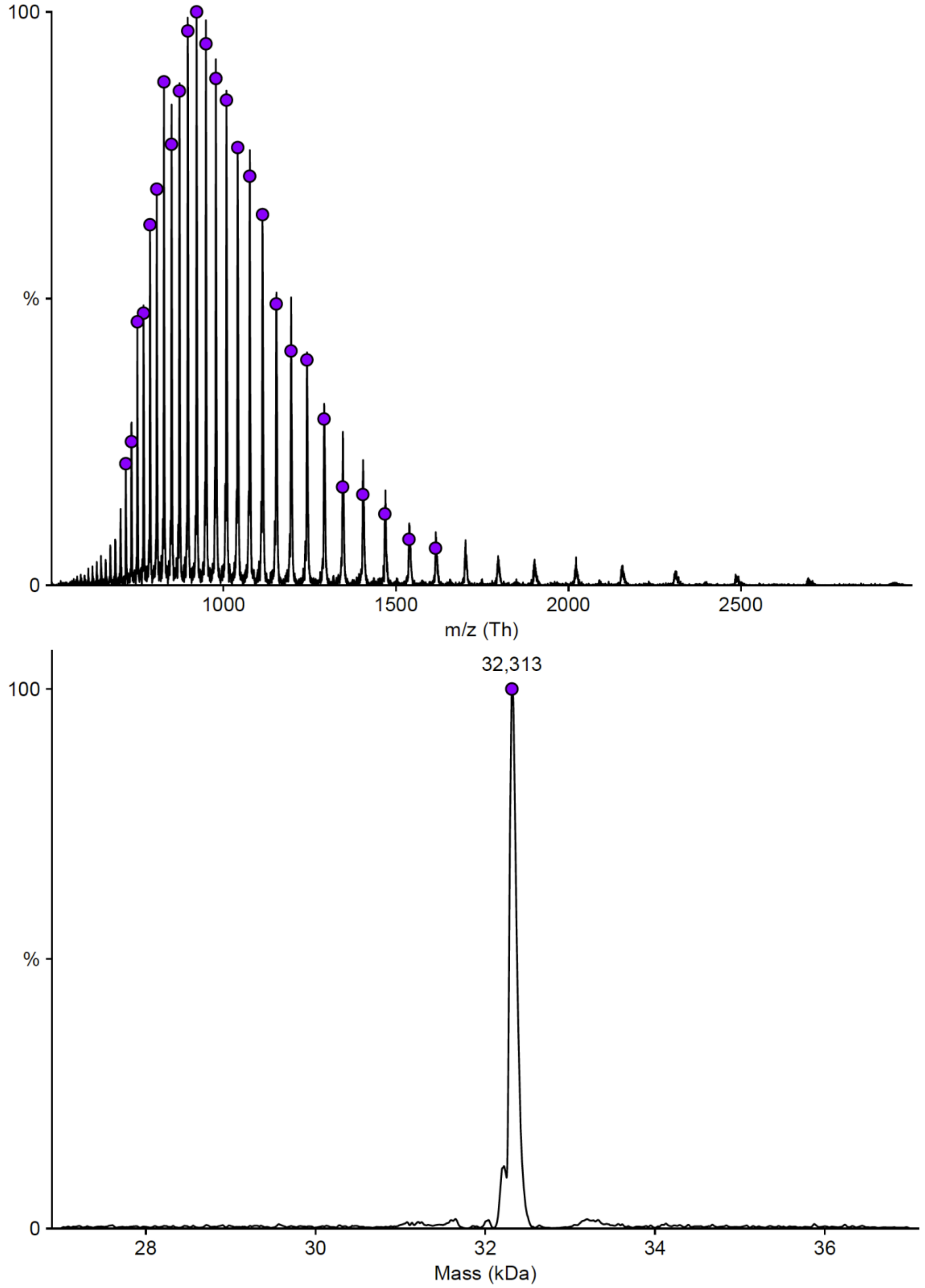

Figure S47. Raw and deconvoluted MS spectrum of 5´-Biotin-**HB8_SC1**, calculated mass: 32296 Da, found mass: 32313 Da.

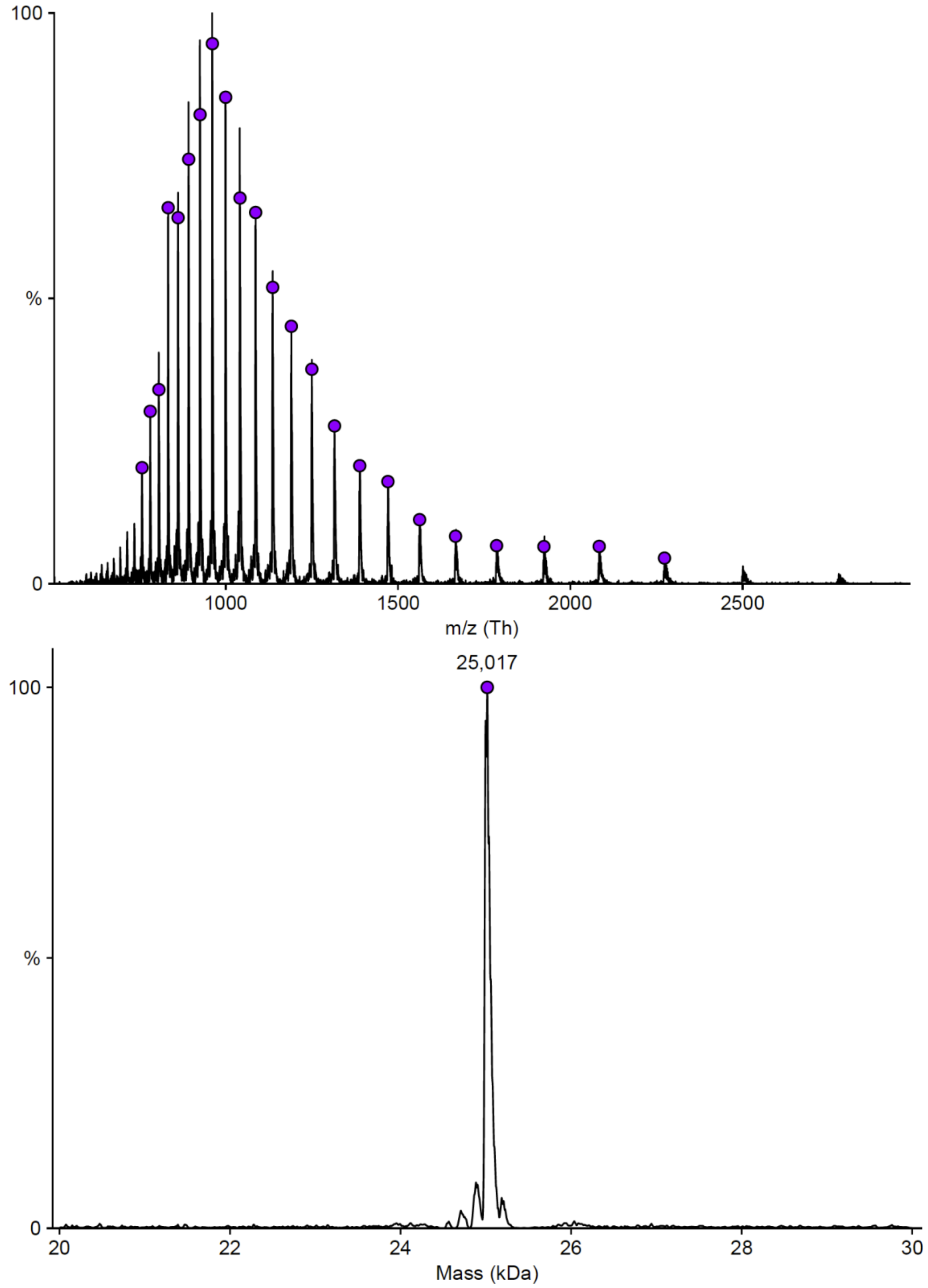

Figure S48. Raw and deconvoluted MS spectrum of 5´-Cy5-**HB8_SC2**, calculated mass: 25004 Da, found mass: 25017 Da.

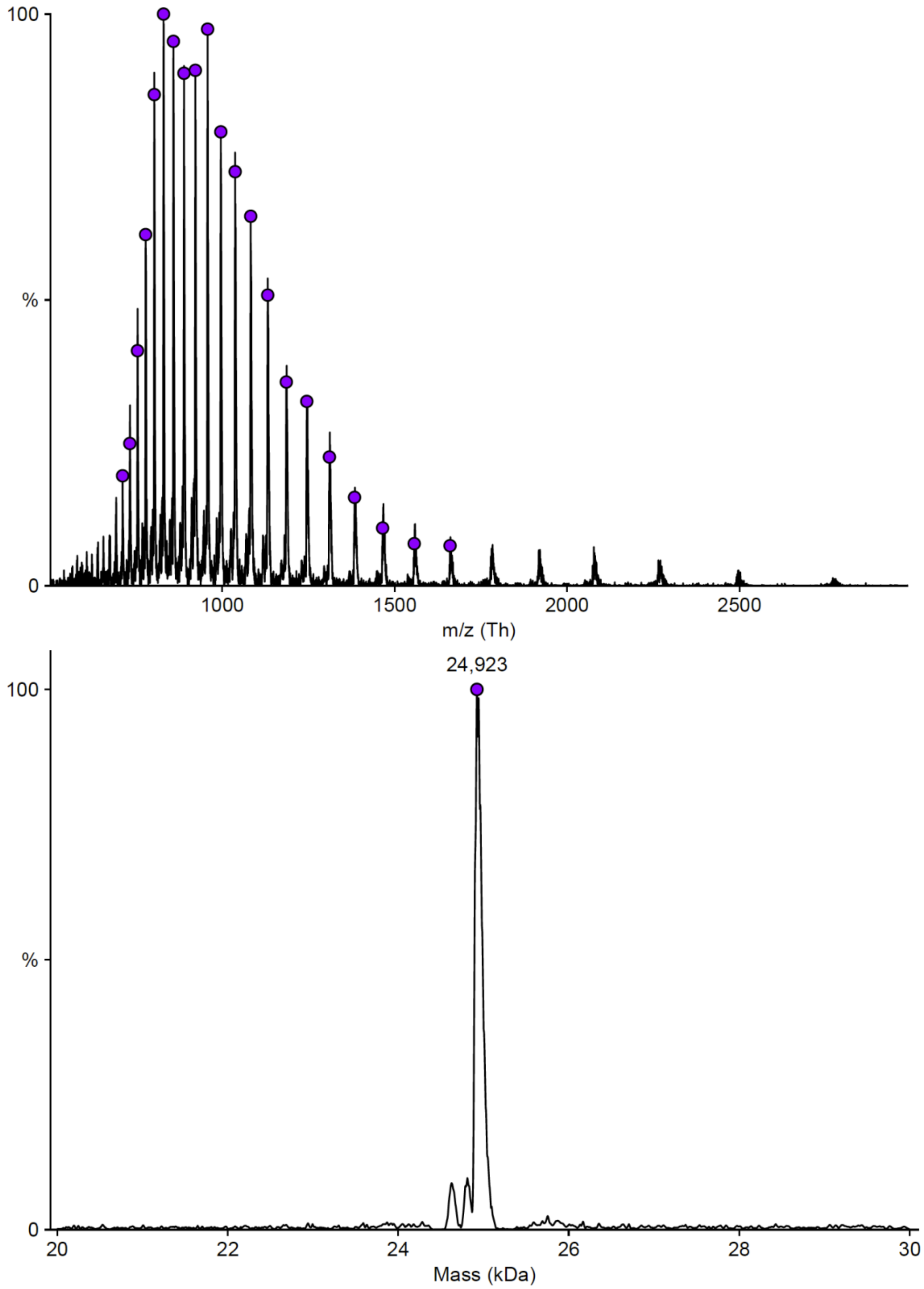

Figure S49. Raw and deconvoluted MS spectrum of 5´-Biotin-**HB8_SC2**, calculated mass: 24908 Da, found mass: 24923 Da.

8) References

1. M. Ondruš, V. Sýkorová, L. Bednárová, R. Pohl, M. Hocek, *Nucleic Acids Res.* **2020**, 48(21), 11982-11993.

2. M. T. Marty, A. J. Baldwin, E. G. Marklund, G. K. A Hochberg, J. L. P. Benesch, C. V. Robinson, *Anal. Chem.* **2015**, 87(8), 4370-4376.

3. P. Nadal, A. Pinto, M. Svobodova, N. Canela, C. K. O´Sullivan, *PLoS One* **2012**, 7(4), e35253.

4. C. A. Schneider, W. S. Rasband, K. W. Eliceiri, *Nature Methods*, **2012**, 9(7), 671–675.

5. M. Martin, *EMBnet J.* **2011***,* 17, 10–12.

6. W. Shen, S. Le, Y. Li, & F. Hu, *PLoS One*. **2016**, 11(10), e0163962.

7. A. Gordon & G. J, Hannon, *FASTX-Toolkit. Hannon Lab.* **2014**, http://hannonlab.cshl.edu/fastx_toolkit

8. S. T. Kramer, P. R. Gruenke, K. K. Alam, D. Xu & D. H. Burke, *Mol Ther Nucleic Acids*. **2022**, 29, 862–70.
